# The oral cavity and intestinal microbiome in children with functional constipation

**DOI:** 10.1038/s41598-024-58642-2

**Published:** 2024-04-09

**Authors:** Monika Kwiatkowska, Marcin Gołębiewski, Marcin Sikora, Ewa Łoś Rycharska, Aneta Krogulska

**Affiliations:** 1 Department of Paediatrics, Allergology and Gastroenterology, Ludwik Rydygier Collegium Medicum in Bydgoszcz, Nicolaus Copernicus University, 87-100 Torun, Poland; 2 Department of Plant Physiology and Biotechnology, Nicolaus Copernicus University, 87-100 Torun, Poland; 3 Interdisciplinary Centre of Modern Technologies, Nicolaus Copernicus University, 87-100 Torun, Poland; 4 Bydgoszcz, Poland

**Keywords:** Microbiology, Gastroenterology

## Abstract

Constipation is a widespread problem in paediatric practice, affecting almost 30% of children. One of the key causal factors of constipation may be disturbances in the homeostasis of the gastrointestinal microbiome. The aim of the study was to determine whether the oral and fecal microbiomes differ between children with and without constipation. A total of 91 children over three years of age were included in the study. Of these, 57 were qualified to a group with constipation, and 34 to a group without. The saliva and stool microbiomes were evaluated using 16S rRNA gene amplicon sequencing. Functional constipation was associated with characteristic bacterial taxa in the fecal microbiota. Statistically significant differences were found at the family level: Burkholderiaceae (q = 0.047), Christensenellaceae (q = 0.047), Chlostridiaceae (q = 0.047) were significantly less abundant in the constipation group, while the Tannerellaceae (q = 0.007) were more abundant. At the genus level, the significant differences were observed for rare genera, including *Christensenellaceae* r-7 (q = 2.88 × 10^−2^), *Fusicatenibacter* (q = 2.88 × 10^−2^), *Parabacteroides* (q = 1.63 × 10^−2^), *Romboutsia* (q = 3.19 × 10^−2^) and *Subdoligranulum* (q = 1.17 × 10^−2^). All of them were less abundant in children with constipation. With the exception of significant taxonomic changes affecting only feces, no differences were found in the alpha and beta diversity of feces and saliva. Children with functional constipation demonstrated significant differences in the abundance of specific bacteria in the stool microbiome compared to healthy children. It is possible that the rare genera identified in our study which were less abundant in the constipated patients (*Christensellaceae r-7, Fusicatenibacter, Parabacteroides, Romboutsia* and *Subdoligranulum*) may play a role in protection against constipation. No significant differences were observed between the two groups with regard to the saliva microbiome.

## Introduction

Constipation is a widespread problem in paediatric practice, which affects almost 30% of the developmental age population^1,2^. These problems persist into adulthood in as many as 25% of cases^3^, and childhood constipation may be a predictor of irritable bowel syndrome in adulthood. Constipation can significantly reduce the quality of life of children and their families^2,4^, and its prevalence is increasing^4^. This increase has been attributed to dietary errors, decreased physical activity, rapidly progressing social and cultural changes, increasing levels of stress and inadequate parental attitudes^4,5^. One of the key causal factors of constipation is believed to be disturbances in the homeostasis of the gastrointestinal microbiome^1,6,7^.

In as much as 90–95% of cases, constipation has a functional basis deriving from a combination of dysfunction in the large intestine, and abnormalities in the pelvic floor and anal sphincter. Thus, functional constipation (FC) can be divided into normal transit constipation (NTC)^8^, slow transit constipation (STC)^9^ and defecation disorders^10^. Unlike defecation disorders, NTC and STC have been found to be associated with a disturbed intestinal microbiome.^10^. However, the causal relationship between such changes in the microbiome and impaired intestinal motility remains poorly understood. Nevertheless, the gut microbiome is known to influence the intestinal peristalsis through various mechanisms, such as changes in intestinal pH^5,11^, butyric acid concentration^1,11^ metabolism of bile acids^12^, neuroendocrine factor production^1,12^, methane production in the intestinal lumen^13^, and the modulation of gene expression^14^. Although it has been shown that the composition of the gastrointestinal microbiome differs significantly between those with constipation and those without^6,7,12,15^, the body of evidence concerning the influence of particular strains on the occurrence of constipation is often contradictory. Fortunately, studies based on bacterial 16S rRNA gene sequencing using specific primers, and DNA sequence analysis in intestinal microbiome taxa have yielded a deeper understanding of the composition of the intestinal microbiome in patients with constipation^16^.

Despite repeated study of the microbiota of the upper and lower gastrointestinal tract, the oral microbiome of constipation patients remains unknown. As saliva constantly flows into the digestive tract, it is possible that the salivary microbiome may influence the development of the gut microbiome. The bacterial compositions of the human oral and intestinal microbiota are closely related, and evidence suggests that studies of the oral microbiome can provide an insight into the intestinal microbiome^17^.

Despite the current standards of disease management, most methods used so far in the treatment of constipation are not very effective. Constipation typically resolves after one year of intensive treatment in approximately 50–60% of children, but persists in the remaining 40–50%^18,19^. In addition, approximately 50% of sick children were found to demonstrate at least one relapse within five years after the resolution of constipation^19^. Such low treatment effectiveness, combined with the growing constipation incidence, drives constant search for novel therapeutic options.

In view of the documented relationship between FC and dysbiosis of the gastrointestinal microbiome^6,7,15^, it has been proposed that influencing the microbiome through the use of probiotics and synbiotics may relieve the symptoms. However, existing findings are inconsistent^20,21^. For such measures to be effective, it is necessary to know the precise composition of the gut microbiome in patients suffering from this disease. Therefore, the aim of the study was to determine whether the oral and gut microbiome differs in children with and without functional constipation. We hypothesized that (i) certain microorganisms detected in oral cavity could be found in stool samples and (ii) community structures both in saliva and stool samples coming from patients with and without constipation would differ.

## Methods

The study was conducted prospectively among patients of the Department of Paediatrics, Allergology and Gastroenterology, between November 1, 2018 and November 30, 2019. A total of 140 children were initially included in the study. However, as 49 failed to provide a complete set of saliva and stool samples, the study included a total of 91 children over three years of age; of these, 57 were qualified to the constipation group, and 34 to the control group (healthy children without constipation). The inclusion and exclusion criteria are presented in Table 1. The characteristics of the two groups are presented in Table 2.

**Table 1:** Inclusion and exclusion criteria for patients in the study and control groups.

Inclusion criteria for patients in the study group	Inclusion criteria for patients in the control group	Exclusion criteria for patients in the study and control groups
Functional constipation diagnosed according to Rome IV criteria Age > 3 years old Consent to participate in the study	Patients without constipation, in whom organic diseases have been ruled out, not under the care of specialized clinics Age > 3 years old Consent to participate in the study	Probiotics in the previous 4 weeks Antibiotic therapy within the last 4 weeks Eating less than 2 h apart when saliva is collected The presence of genetic syndrome, psychomotor developmental delay, chronic encephalopathy, spina bifida, Hirschsprung's disease, anatomical defects of the lower gastrointestinal tract (including anal stenosis), celiac disease, disease of the inflammatory bowel disease, infectious colitis

**Table 2:** Characteristics of the study and control groups.

Parameter	Children with FC n = 57 (100%)	Children without FC n = 34 (100%)	P
Age, years			
Mean ± SD	8.920 ± 3.772	11.455 ± 4.056	0.029
Sex, n (%)			> 0.05
Boys	30 (50.9)	18(52.9)	
Girls	28 (49.1)	16 (47.1)	
Residence, n (%)			> 0.05
City	36 (63.2)	20 (58.8)	
Country	21 (36.8)	14 (41.2)	
Parents’ education, n (%)			> 0.05
Mother			
Primary education	11 (19.3)	7 (20.6)	
Secondary esucation	17 (29.8)	12 (35.3)	
Higher education	29 (50.9)	15 (44.1)	
Father			
Primary education	14 (24.6)	11 (32.4)	
Secondary esucation	19 (33.3)	12 (35.3)	
Higher education	24 (42.1)	11 (32.4)	

The research materials comprised a questionnaire constructed for the study, together with a diet composition assessment and microbiome assessment. The questionnaire concerned sociodemographic data, reported ailments, physical activity, ways of spending free time, eating habits, family interview.

### Sample collection

**Figure 1:**
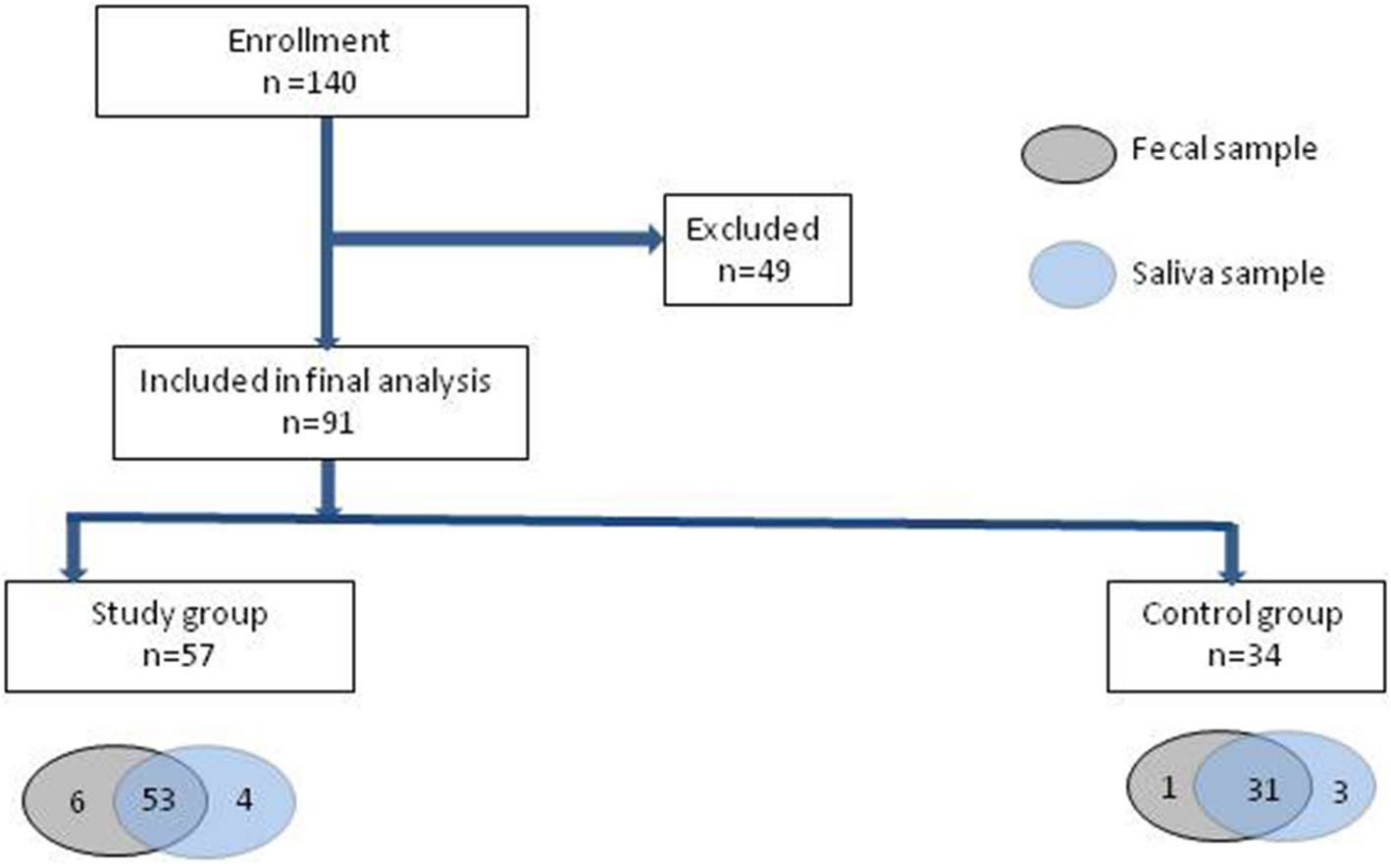
Study design. Flow chart depicting steps involved in patient selection and tests in this study.

Saliva and stool samples were collected from the children on one occasion for microbiome assessment: each participant provided 50 mg of stool matter and 3 ml of saliva. All samples were stored at − 80 °C until DNA isolation. The recruitment and sample collection procedures are depicted in Fig. 1. As seven saliva samples and five fecal samples were of poor quality, only 84 saliva samples and 86 stool samples were included in the analysis (170 samples in total).

### Microbiome diversity assessment

DNA isolation, library preparation, quality assessment and quantification were performed as described previously^22^. Sequencing was performed using a MiSeq sequencing kit v.3, for 600 cycles on a MiSeq apparatus (Illumina) at CMIT NCU.

### Statistical analysis

Statistical analysis of clinical variables was performed using the SPSS software package (IBM). For the quantitative differences, descriptive statistics were calculated and the two groups were compared using the Mann–Whitney test. For the nominal and ordinal variables, the data was analysed using contingency tables and chi-square tests. Significant relationships were noted for *p* < 0.05.

The differences in the abundance of taxa were assessed by ANOVA (aov function in R); the normality of the data was checked with the Shapiro–Wilk test (shapiro.test), and the homogeneity of variance with Levene's test (levene.test from lawstatpackege). If these assumptions were not met, the Kruskal–Wallis test (kruskal.test) was used. *p*-values were corrected for multiple comparisons using the Benjamini–Hochberg FDR (p.adjust). Categorical clinical data was analysed using two-tailed Fisher’s exact test in R (fisher.test); the level of significance was assumed to be *p* = 0.05^23^.

### Bioinformatic analysis

Reads were denoised and assembled, then chimaeras were identified and removed using dada2 R package^24^, as described earlier^22^.

Briefly, the sequencing reads were denoised, merged and assessed for the presence of chimeras in dada2; and then classified using the SILVA database. OTUs were constructed and OTU shared tables were constructed using the Mothur program. The analyses were performed in the R program using the *vegan* and *GUniFrac* packages. An unweighted UniFrac distance matrix was generated using the *GUniFrac* function from the OTU table using the Relaxed Neighbor Joining algorithm. The unconstrained ordination was performed using non-metric multidimensional scaling (NMDS) implemented in the metaMDS function of *vegan*. The significance of the grouping was tested by the PERMANOVA method (adonis function) using 999 permutations, dbRDA was performed on an unweighted UniFrac distance matrix. The significance of the dbRDA model was tested using a permutational test (anova.rda) using 999 permutations.

Characteristic taxa were identified using sPLS-DA analysis implemented in the splsda function of mixOmics R package^25^. The protocol used was described on the mixOmics webpage^26^.

### PICRUSt2 prediction of metabolic potential

Metabolic potential of bacterial communities thriving in samples under study was predicted using PICRUSt2^27^ using default parameters. Differentially abundant features (KEGG Orthologies and pathways) were identified with DESeq2.

The research was conducted with the consent of the local Ethics Committees of the Institutional Review Board of CM NCU (KB 748/2018). The study was performed in accordance with relevant guidelines and regulations, as well with the principles of the Declaration of Helsinki. Written informed consent was obtained from the parents of each patient or their legal guardians, prior to their enrolment.

The datasets generated and/or analysed during the current study are available in the NCBI's SRA repository, under BioProject accessions PRJNA925675 (saliva samples) and PRJNA925436 (fecal samples).

The experimental protocols were approved by the local Ethics Committees of the Institutional Review Board of CM NCU. All methods were carried out in accordance with relevant guidelines and regulations.

## Results

### Alpha-diversity patterns

**Figure 2:**
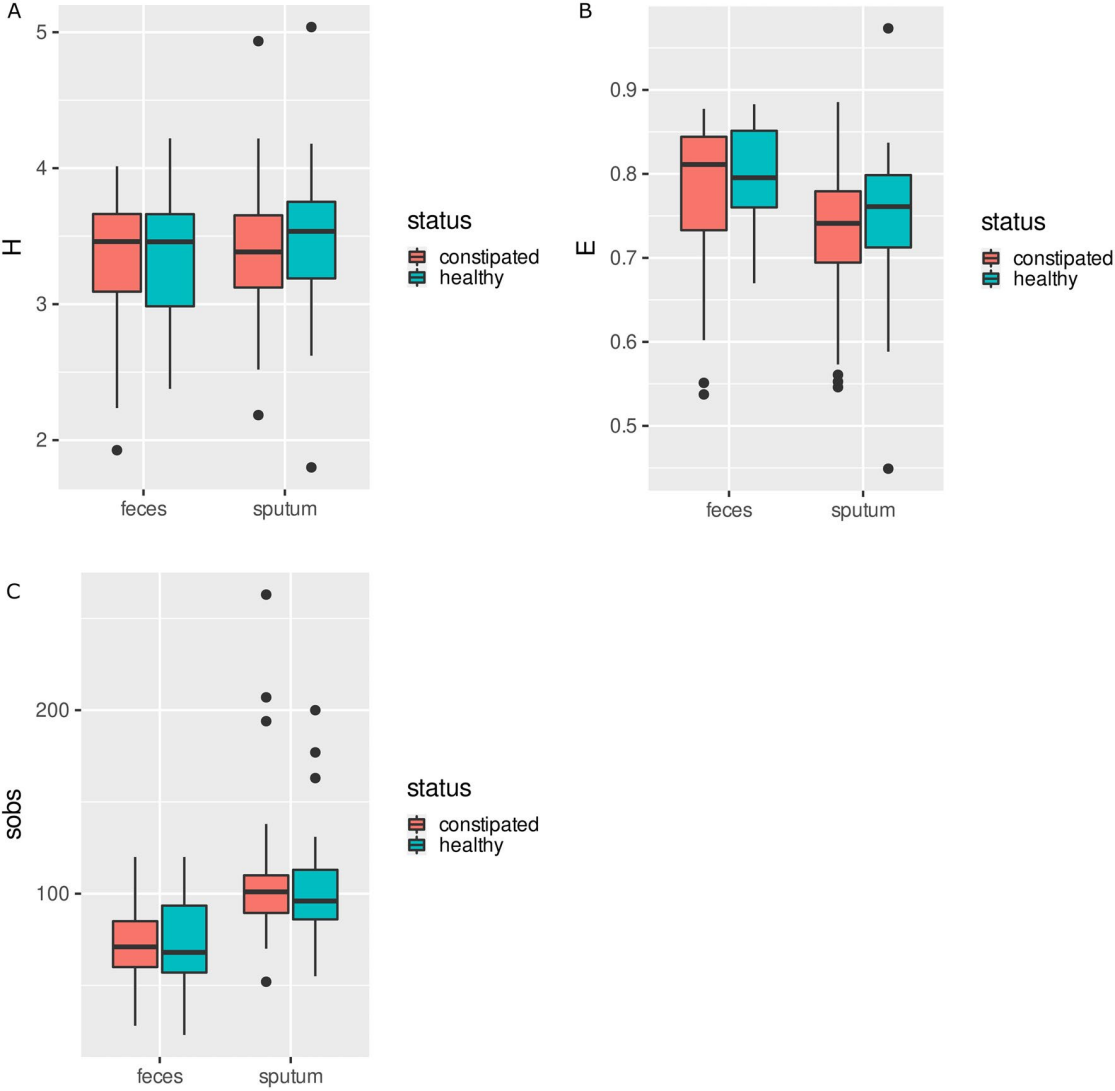
(**A**), (**B**), (**C**) Alfa – diversity—sputum and feces microbiome of constipated children vs healthy children. (**A**) Shannon diversity index. (**B**) Shannon evennes index. (**C**) Sob index. Meron squre—constipated. Blue square—healthy.

The bacterial communities demonstrated significantly higher species richness and evenness in the fecal samples than the saliva (Kruskal–Wallis, *p* = 6.537e−14 and *p* = 3.913e−6, respectively); however, no difference in diversity was observed in the FC group nor in the non-FC group (Shannon’s H’, Kruskal–Wallis, *p* = 0.722). No significant differences in species richness, diversity and evenness were found between the group with FC and the group without FC, regardless of material analyzed Fig. 2.

### Beta-diversity

**Figure 3:**
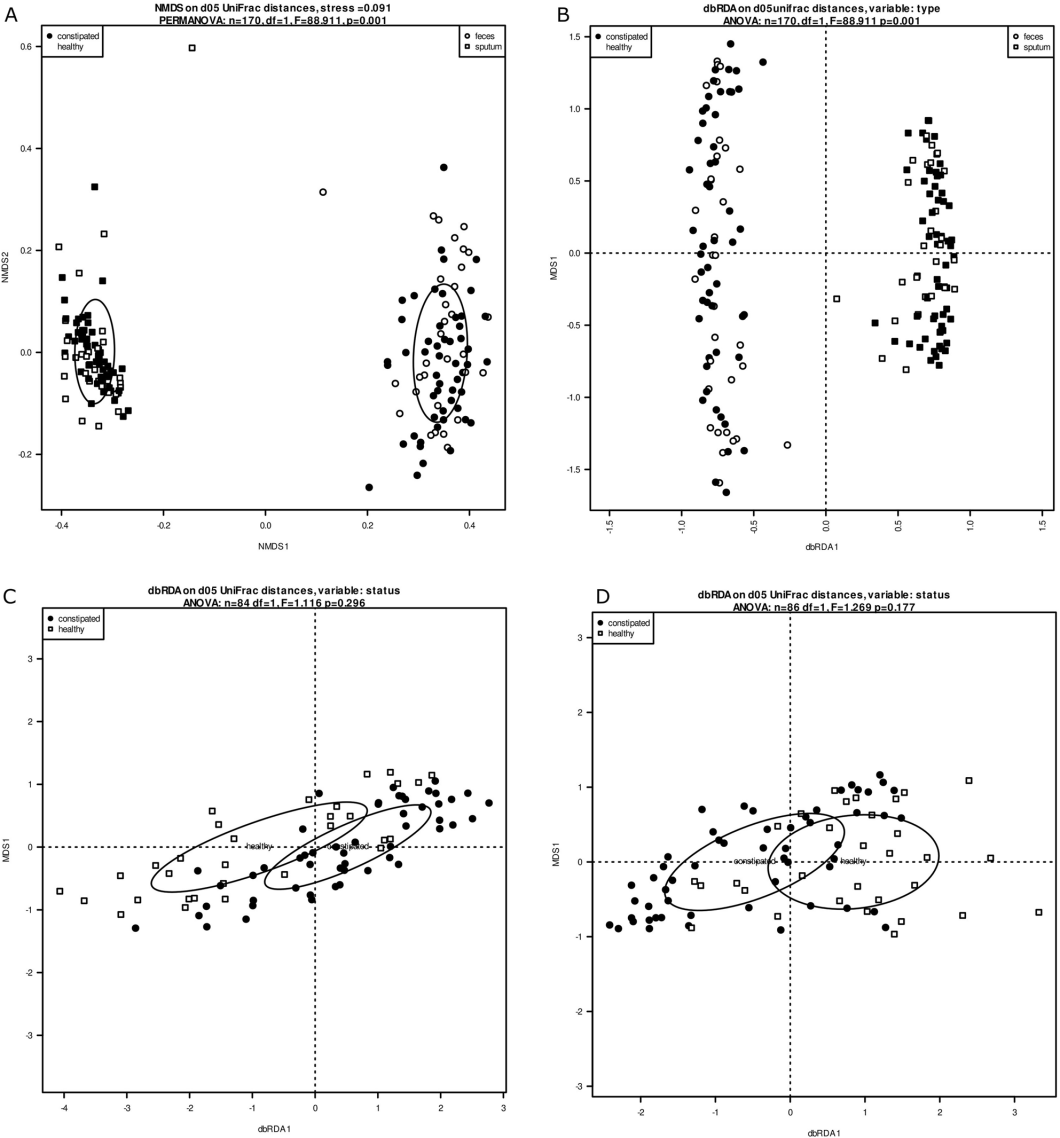
(**A**)–(**D**) Beta—diversity—scommunities in saliva and fecal samples grouped according to their origin and studied groups. (**A**) NMDS plot (PERMANOVA, n = 170, R^2^ = 0.346, F = 88.911, p = 0,001). (**B**) Dbrda (n = 170, F = 88.911, p = 0,001). (**C**) PERMANOVA- fecal samples (n = 84, R^2^ = 0.031, F = 1,11, p = 0,296). (**D**) PERMANOVA (n = 86, R^2^ = 0.013, F = 1.269, p = 0,177)- saliva samples. Filled circle: Feces constipated. White circle: Feces healthy. Filled square: Sputum constipated. White square: Sputum healthy.

There were significant differences in grouping of communities in saliva and fecal samples according to their origin on an NMDS plot (PERMANOVA, n = 170, R^2^ = 0.346, F = 88.898, *p* = 1e−4; Fig. 3A) which was further corroborated by dbRDA analysis (n = 170, F = 88.898, *p* = 1e−4; Fig. 3B). Therefore, the two kinds of samples were analyzed separately. No significant differences between communities coming from FC and non-FC samples were found both in fecal and saliva samples (PERMANOVA, n = 84, R^2^ = 0.031, F = 1.11, *p* = 0.296; Fig. 3C), PERMANOVA, n = 86, R^2^ = 0.013, F = 1.269, *p* = 0.177; Fig. 3D).

### Taxonomic structure

**Figure 4:**
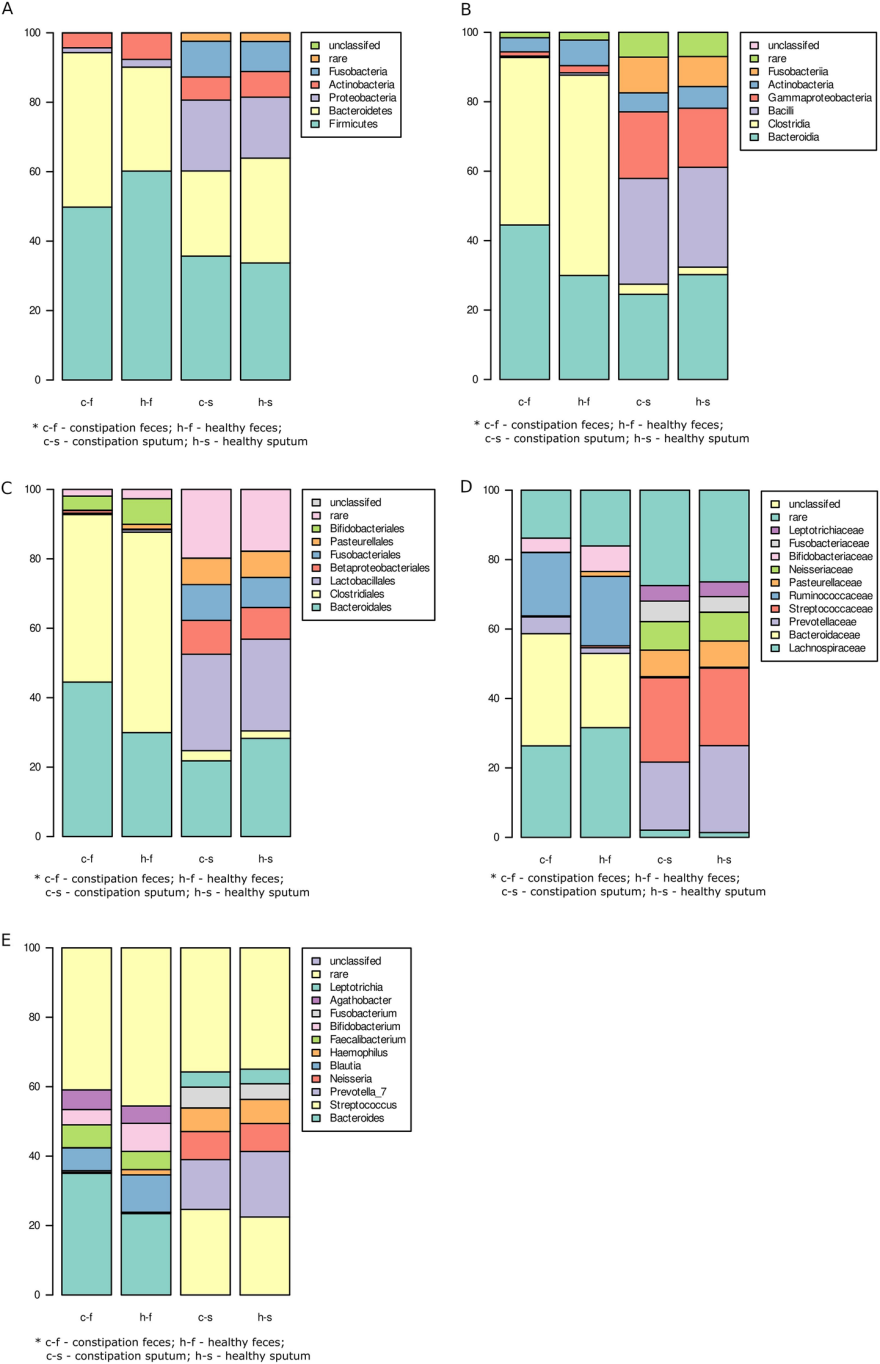
(**A**)–(**E**). Taxonomic analysis of the saliva and stool microbiome including phyla, classes, orders, families and genera. (**A**) Phyla. (**B**) Classes. (**C**) Orders. (**D**) Families. (**E**) Genera. c-f—constipation feces; h-f—healthy feces; c-s—constipation saliva; h–s—healthy saliva.

Conspicuous differences were found between the saliva and feces microbiomes, regardless of the taxonomic level (Fig. 4A,B). The libraries derived from the fecal samples were dominated by sequences classified as Clostridia and Bacteroidia, with a minor admixture of Actinobacteria, while those generated from the saliva samples were abundant in sequences affiliated with Bacilli and Gammaproteobacteria. At the genus level, the most abundant taxa in the fecal samples were absent from the saliva samples, and vice versa. The most abundant genera was *Bacteroides* in the fecal libraries, and *Streptococcus* in the saliva libraries.

In the saliva samples, no significant differences at any taxonomic level were found between the healthy and constipated groups regarding the abundance of taxa (*p* > 0.05) (Fig. 4A,B).

However, in the stool samples, Bacteroidetes was found to be more abundant in children with constipation. The FC group also demonstrated a significantly lower abundance of Firmicutes, Proteobacteria and Actinobacteria than the non-FC group; however, these differences were not statistically significant (*p* > 0.05) (Fig. 4A). At the class level, the constipation group demonstrated a greater abundance of Bacteroidia, and lower levels of Clostridia, Actinobacteria, and Gammaproteobacteria; again, however, these differences were not statistically significant (*p* > 0.05) (Fig. 4B). At the order level, Bacteroidales were more abundant in the constipation group and Clostridiales, Pasteurellales and Bifidobacteriales were less abundant. The differences were not statistically significant (*p* > 0.05) (Fig. 4C).

At the family level, *B*acteroidaceae and Prevotellaceae were more abundant in the constipation group, while Lachnospiraceae, Ruminococcaceae, and Bifidobacteriaceae were less abundant. In addition, statistically-significant differences were found for rare families:

Burkholderiaceae (Kruskal–Wallis, Benjamini–Hochberg corrected for multiple comparisons, q = 0.047), Christensenellaceae (q = 0.047), Clostridiaceae (q = 0.047) were significantly less abundant in the constipation group, while the Tannerellaceae (q = 0.007) were more abundant (Fig. 4D).

At the genus level, the constipation group demonstrated a greater abundance of *Bacteroides*, *Faecalibacterium* and *Agatobacter*, and a lower level of *Blautia* and *Bifidobacterium.* The only significant differences were observed for rare genera, including *Christensenellaceae* r-7 (q = 2.88 × 10^−2^), *Fusicatenibacter* (q = 2.88 × 10^−2^), *Parabacteroides* (q = 1.63 × 10^−2^), *Romboutsia* (q = 3.19 × 10^−2^) and *Subdoligranulum* (q = 1.17 × 10^−2^). All were less abundant in children with constipation (Fig. 4E). The compositions of the gut microbiomes of the two patient groups are compared in Table 3.

**Table 3:** Taxonomic changes in the gut microbiome in children with FC compared to children without FC.

Taxonomy	Children with FC vs children without FC			
	More	Less		No difference
Phyla	Bacteroidetes	Firmicutes	Proteobacteria	Actinobacteria
Classes	Bacteroidia	Clostridia	Gamma-proteobacteria	Actinobacteria
Orders	Bacteroidales	Clostridiales	Pasteurellales	Bifidobacteriales
Families	Bacteroidaceae Prevotellaceae Tannerellaceae^a^	Lachnospiraceae Ruminococcaceae Chrystensenellaceae^b^ Chlostridiaceae^#1^	Burkholderiaceae^c^ (Beta-proteobacteria)	Bifidobacteriaceae
Genera	Bacteroides Parabacteroides^d^ Romboutsia^e^	Faecalibacterium Blautia Chrystensenellaceae r-7^f^ Fusicatenibacter^g^ Subdoligranulum^h^	Agatobacter (Alfa-proteobacteria)	Bifidobacterium

^a^q = 0.007, stastically sagnificant increase; ^b^q = 0.047, stastically sagnificant decline; ^c^q = 0.047, stastically sagnificant decline; ^d^q = 0.016, stastically sagnificant decline; ^e^q = 0.031, stastically sagnificant decline; ^f^q = 0.028, stastically sagnificant decline; ^g^q = 0.028, stastically sagnificant decline; ^h^ q = 0.011, stastically sagnificant decline.

Some of the bacterial species present in the saliva and stool samples from the constipation group were not present in those of the non-constipation group (Table 4).

**Table 4:** Co-occurrence of bacteria in saliva and stool in children with FC vs children without FC.

Co-occurring bacteria	
Children with FC n = 57 (100%)	
ASV 6 (Streptococcus)	Rho = 0.29; p = 0.038
ASV 180 (Streptococcus)	Rho = 0.32; p = 0.003
ASV 243 (Lactobacillus)	Rho = 0.46; p = 5 × 10^–4^
ASV 505 (Lactobacillus)	Rho = 0.45; p = 7 × 10^–4^
ASV290 (Haemophilus)	Rho = 0.35; p = 0.011
ASV 991 (Lactobacillus)	Rho = 1; p = 1
ASV 1462 (Lactobacillus)	Rho = 1; p = 1
Children without FC n = 34 (100%)	
123 (Haemophilus)	Rho = 0.39; p = 0.026
ASV 299 (Dialister)	Rho = 0.47; p = 0.006
ASV 307 (Lactococcus)	Rho = 0.47; p = 0.005
ASV314 (Bifidobacterium)	Rho = 0.52; p = 0.002
ASV 603 (Haemophilus)	Rho = 0.72; p = 0.000

*ASV* amplicon sequence variants.

**Figure 5:**
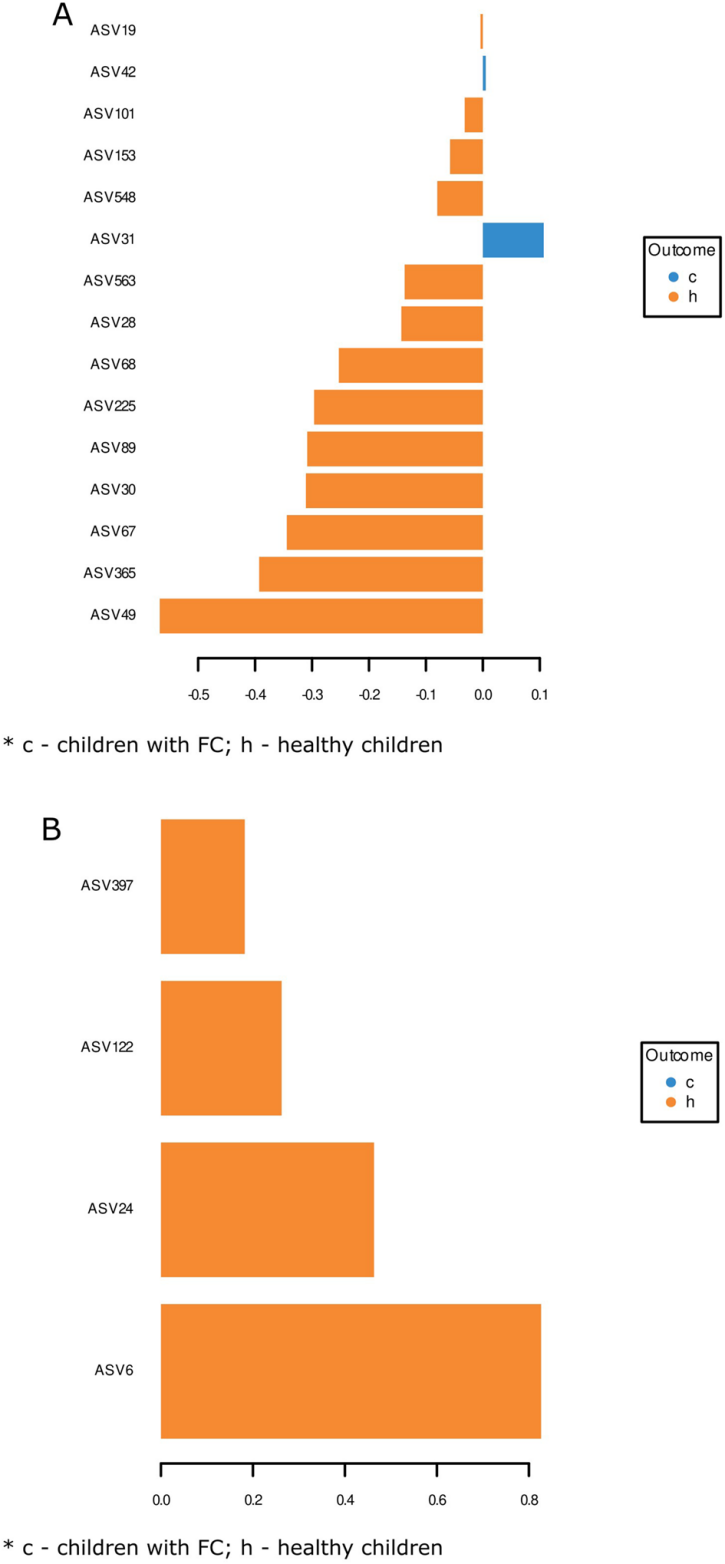
(**A**), (**B**) Amplicon sequence variants in stool and saliva microbiomes in children with FC vs without FC. (**A**) Stool microbiome in children with FC vs healthy children. (**B**) Saliva microbiome in children with FC vs healthy children. Yellow circle: h. Blue circle: c. c-children with FC; h-healthy children.

The most characteristic sequence variants were ASV (amplicons sequence variant) 31 (*Parabacteroides*) for the stool microbiome of constipated patients, and ASV 49 (*Subdoligranulum*) and ASV 365 for the stool microbiome of the non-constipated group (Fig. 5A).

The most characteristic sequence for the saliva microbiome in healthy subjects was the ASV6 (*Streptococcus*) variant (Fig. 5B); however, no ASVs in the saliva appeared to differentiate constipated and control patients.

### Analysis of metabolic potential

**Figure 6:**
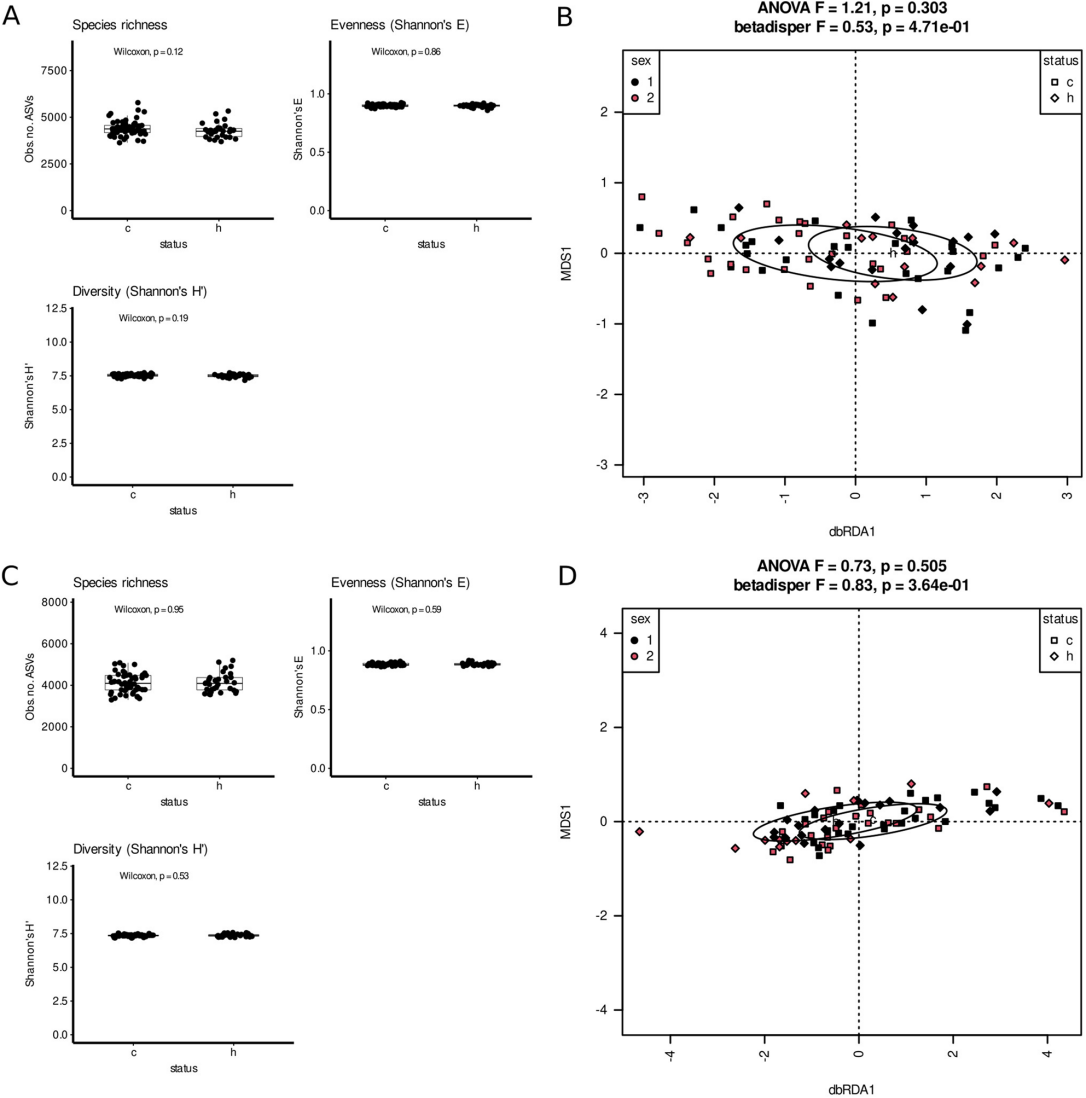
(**A**)–(**D**) Alpha- and beta-diversity analysis of PICRUSt2-predicted metabolic potential of bacterial communities. (**A**) Alpha-diveristy in saliva samples, (**B**) dbRDA analysis based on Morisita-Horn distance matrix of saliva samples, (**C**) alpha-diversity in fecal samples, (**D**) dbRDA analysis based on Morisita-Horn distance matrix of fecal samples. c-children with FC. h-healthy children.

There were no significant differences between sets of functions predicted to be encoded in genomes of bacteria thriving in constipated and healthy patients’ feces and sputum, neither in terms of alpha- nor beta-diversity (Fig. 6). However, we identified differentially abundant functions and pathways. In case of feces functions involved in benzoate metabolism (catechol pathway) were less frequently encountered in genomes of bacteria from constipated patients, while functions involved in antibiotics resistance, short fatty acids production or competence were more frequent in organisms from constipated patients (Table 1 Suppl, Table 2 Suppl). In sputum genes involved in chlorophyll metabolism and biosynthesis were more abundant in genomes of bacteria from constipated patients, while functions related to sugar metabolism/catabolism were more frequent in organisms living in healthy patients (Table 3 Suppl, Table 4 Suppl).

### Analysis of diet and exercise

No statistically significant differences in diet composition or physical activity were found between the two groups. The results are shown in Table 5 Suppl and Table 6 Suppl.

## Discussion

The disturbances in microbiome homeostasis may constitute a modifiable factor influencing the development and course of constipation in children^6,7^.

Few studies have analysed the hypothesis that the intestinal microbiome has an influence on constipation, even fewer have been conducted in the paediatric population^1,6,7^, and their findings are varied or even contradictory^1,6,15,28^. Furthermore, no studies evaluating the composition of the upper gastrointestinal microbiome and its role in the pathogenesis of constipation in children have been published to date. It is also unclear how similar the microbiomes of the upper and lower gastrointestinal tract are, and which bacterial taxa are shared between them.

To address this knowledge gap, the present study provides new data on the intestinal microbiome in Polish children with constipation; however, unlike previous studies, it also compares the findings with the microbiome of the saliva in this group.

No significant differences in diversity, evenness or species richness were observed in the saliva microbiome of the two groups of children. While relatively little is known about the saliva microbiome, its composition has been found to influence oral diseases such as tooth decay and recurrent aphthous stomatitis^29,30^, as well as systemic diseases such as rheumatoid arthritis^31,32^. However, no studies have evaluated the relationship between the saliva microbiome and constipation.

Regarding the relationship between the gut microbiome and the development of disease, we assumed that the gut and salivary microbiomes may differ between children with FC and those without. However, no such differences were observed between the two groups, neither in the salivary microbiome, nor in that of the lower GI. This can be explained by the heterogeneity of human microbiome, making identification of general trends difficult in relatively small groups, such as those included in the present study. However, in spite of the lack of significance of dbRDA and PERMANOVA models, we were able to identify organisms whose abundance differed in constipated and healthy patients, suggesting that they may play a role in the pathogenesis of constipation. Although no statistically-significant differences in the abundance of high-level taxa were observed between the two groups, our results indicate that the most characteristic sequence for saliva in both FC and non-FC children was ASV6, classified as *Streptococcus*. This has been described previously^33–36^.

Much more research has been devoted to analysing the stool microbiome than the salivary microbiome. In the present study, no differences in alpha-diversity were observed in the stool microbiome between the two groups of children. Similar results were obtained by de Meij et al. who compared the stool microbiomes of 76 children with FC with those of 61 children without FC^7^. However, our findings indicate no significant differences in community structure and slight differences in taxonomic composition between the stool microbiomes of the two groups. Previous studies have also noted differences in the composition of the stool microbiome between patients with constipation and those without^1,6,12,15,28^.

The present study found a smaller abundance of Firmicutes in the stool samples from the constipation group compared to the non-constipated group. In addition, statistically-significant differences were observed for rare families and genera, such as members of Christensenellaceae (Firmicutes, first detected in the stool of a non-constipated subject), Clostridiaceae and *Christensenellaceae r-7*, *Fusicatenibacter* and *Romboutsia*. These are similar to previous findings^6,15,37,38^.

Other studies suggest that the Christensenellaceae have may have beneficial effects on their host. Their abundance tends to be lower in patients with irritable bowel syndrome, obesity, elevated serum triglyceride levels, and with metabolic syndrome^39–42^, and higher in those with normal blood pressure^42^. While a better understanding of the Christensenellaceae may offer hope for new treatment options, the mechanism of its action currently remains to be elucidated^43,44^.

An analysis of the stool microbiome of 57 adult patients based on traditional bacterial culture revealed a lower abundance of Firmicutes (mainly Lactobacillus and Clostridium) in patients with constipation compared to patients without^15^; elsewhere, a greater abundance of Firmicutes in the stools of constipated patients were also observed^28^. Assuming that the presence of Firmicutes increases the rate of peristalsis, it can be hypothesized that their absence may promote the occurrence of constipation^15^. Nevertheless, Zhu et al.^1^ report a greater abundance of Firmicutes in children with constipation compared to those without; however, this study only included a specific group of patients, i.e. obese children (BMI > 95pc), and this could have influenced the obtained results. Obesity is known to be associated with a specific composition of the intestinal microbiome, with a greater abundance of Firmicutes compared to Bacteroides and faster peristalsis compared to children with normal body weight^12^.

Our present findings also indicate a greater abundance of Bacteroidetes representatives in the stool microbiome in children with FC compared to those without, which is consistent with previous results^12,28^. In addition, statistically significant differences in the presence of the rare genus *Parabacteroides* were found between the two groups. Parthasarathy et al.^12^ report that an increased abundance of Bacteroidetes representatives is associated with a slower passage of the intestinal contents through the gastrointestinal tract.

In the present study, although the abundance of Actinobacteria was insignificantly lower in children with FC, no differences in abundance were found at lower taxonomic levels.

The evidence regarding the significance of *Bifidobacterium*, a genus belonging to the Actinobacteria, in patients with constipation is not unequivocal^1,6,28^. Our results showed lower abundance of *Bifidobacterium* in patients with constipation. As these bacteria demonstrate monosaccharide catabolism, the final metabolites of which are the postbiotics acetate, lactate and ethanol, these compounds are commonly found in the stools of healthy people^45,46^. Due to their beneficial health effects, the bacteria are also used as probiotics^47^.

Our present findings also indicate a significantly lower abundance of Gamma-proteobacteria representatives in the constipation patients compared to the healthy group. In addition, a significantly lower abundance of the Burkholderiaceae family was observed in children with constipation than those without. Although no studies have examined the significance of this family in constipation, some research has confirmed it plays a role in cancer in adult patients; for example, the increased presence of *Burkholderia cepacia* in the gastrointestinal tract of patients receiving immunotherapy based on CTLA-4 (T cell-associated antigen 4) was associated with stronger therapeutic effects and lesser undesirable side effects, such as colitis^48^.

Results of predicted metabolic potential analysis suggest that there are only minor differences between sets of functions encoded by genomes of organisms thriving in constipated and healthy patients. This is caused by large number of core genes present in virtually each bacterium. Therefore, our search focused on differentially abundant features. The greater frequency of genes involved in benzoate metabolism in feces of healthy patients suggests that either these patients consume greater amounts of benzoate or bacterial community with greater capability to metabolize this compound, being frequently used as a food preservative, is involved in preventing constipation. Currently, no reports exist of the role of benzoate in constipation etiology; however, it is known to influence the human gut microbiome^49^. In case of sputum the increased frequency of chlorophyll metabolism/catabolism involved functions in genomes of constipated patients might stem from increased consumption of raw plants frequently thought to be mild laxatives.

Further research will provide a more thorough understanding of the studied bacteria, which may support their use as a primary biomarker in patients with constipation.

Our findings reveal significant differences between the saliva and stool microbiomes, and hence probably also between those of the mouth and the large intestine, both in children with constipation and those without. Thus, the saliva demonstrated greater species richness and less microbiome uniformity compared to the stool. The oral cavity is directly exposed to a number of external factors, and is characterised by diverse physicochemical conditions; this creates opportunities for the existence of a greater variety of inhabiting microorganisms and their uneven distribution^50^.

Although few studies have compared the salivary and stool microbiomes, Tsuda et al. report no significant differences in alpha-diversity between the two in a group of 45 adult patients^23^. They propose that swallowing and intestinal transit result in a natural movement of a large part of the bacteria from the oral cavity to the lower parts of the digestive tract. Despite the length of the passage and the variable physicochemical conditions of the gastrointestinal tract, such as low gastric pH, the presence of digestive enzymes and active immune response, it has been shown that the species of microorganisms detected in the oral cavity and stool coincided in approximately 45% of subjects. The coexistence of bacteria in the upper and lower gastrointestinal tract in infants, children and adults was also confirmed by Yatsunenko et al^51^.

Our present findings indicate that two studied groups of children demonstrate different taxonomical overlaps between the two microbiomes, and that this overlap includes different species of bacteria. Constipation is known to be associated with stool retention in the intestine, which causes changes in the physicochemical conditions of the gut, e.g. in the pH range; this promotes the multiplication and development of different bacteria compared to physiological conditions, and consequently, higher concentrations of their metabolites^8,11^. This leads to a “vicious circle” that sustains changes in the microbiome.

The present study is the first to examine the relationship between the microbiomes of the saliva and the stool in children with functional constipation. One of its key strengths is that the two groups of children were recruited using similar quantitative and qualitative criteria, i.e. they demonstrated comparable consumption of fibre, water, minerals and kcal in the diet, and took part in similar levels of physical activity. Hence, the demonstrated disturbances in microbiome homeostasis were of fundamental importance in the development of constipation in the studied children, and did not result from other factors that potentially affect the microbiome (such as diet or physical activity). Another strength of the study is that the two groups of patients had similar BMI. Obesity is known to cause differences in microbiome composition, regardless of the presence of an abnormal bowel movement pattern.

Another advantage of the study is that it is the first such analysis to assess both the composition of the upper and lower gastrointestinal tract microbiomes and the significance of their role in the pathogenesis of constipation. Moreover, no studies evaluating the coexistence of bacteria in saliva and stool in children with constipation have been published so far: this is only the third published study to analyse the relationship between the composition of the gut microbiome and physical activity, and the first to assess this relationship in the paediatric population. Most importantly, the study uses a methodology for assessing the microbiome which is based on 16 srRNA, which allows for accurate identification of microorganisms in the saliva and stool regardless of their ability to grow under laboratory conditions, down to the level of ASVs.

However, the study also has some limitations: a relatively small group of participants were recruited, particularly the non-constipated group, and age variation was found between the groups, with the children in the study group being younger than those in the control group. It should be emphasized, however, that the identified age differences did not significantly affect the obtained results, because it is known that the microbiome is intensively shaped up to the age of three years, and then remains relatively stable^52^; these changes were taken into account during study group selection (all children were at least three years of age).

Our findings may be used to modify existing algorithms for the treatment of constipation and to identify potential indications for treatment in children with functional constipation, possibly with the use of individualized probiotic therapy. However, further research is needed to accurately determine the composition of the microbiome in the paediatric population affected by this disease.

Children with functional constipation demonstrated significant differences in abundance of specific bacteria in their stool microbiome compared to healthy children. We suggest that rare generate identified in our study as being less abundant in constipated patients (*Christensellaceae r-7, Fusicatenibacter, Parabacteroides, Romboutsia and Subdoligranulum*) might be involved in protection against constipation. No significant differences were observed between the two groups of children with regard to the saliva microbiome.

### Supplementary Information

Supplementary Table 1.

Supplementary Table 2.

Supplementary Table 3.

Supplementary Table 4.

Supplementary Table 5.

Supplementary Table 6.

## Data Availability

All data relevant to the study are included in the article or uploaded as supplementary information. In addition, the datasets used and/or analyzed during the current study are available from the corresponding author on reasonable request.
