# Supplementary material for: The oral cavity and intestinal microbiome in children with functional constipation
Source: Sci Rep. 2024 Apr 9;14:8283. doi: 10.1038/s41598-024-58642-2 (PMC11004141; doi:10.1038/s41598-024-58642-2)
Supplement: Supplementary file 1 — Supplementary Table 1. [file 41598_2024_58642_MOESM1_ESM.docx]

|  | Row.names | baseMean | log2FoldChange | lfcSE | stat | pvalue | padj | fun | desc |  |
| --- | --- | --- | --- | --- | --- | --- | --- | --- | --- | --- |
| 10 | K00638 | 14,80050812 | 3,504181521 | 0,541974221 | 6,46558708 | 1,01E-10 | 3,36E-07 | K00638 | catB | chloramphenicol O-acetyltransferase type B [EC:2.3.1.28] |
| 156 | K13921 | 12,52835994 | 3,139985592 | 0,543191676 | 5,780621708 | 7,44E-09 | 1,25E-05 | K13921 | pduQ | 1-propanol dehydrogenase |
| 122 | K10241 | 7,705098103 | 2,922856199 | 0,509073559 | 5,741520348 | 9,38E-09 | 1,25E-05 | K10241 | cebF | cellobiose transport system permease protein |
| 107 | K08358 | 13,70558748 | 2,616242764 | 0,541614894 | 4,830448337 | 1,36E-06 | 0,000454 | K08358 | ttrB | tetrathionate reductase subunit B |
| 126 | K10873 | 7,169058092 | 2,571139392 | 0,503898369 | 5,10249596 | 3,35E-07 | 0,000199 | K10873 | RAD52 | DNA repair and recombination protein RAD52 |
| 58 | K04844 | 74,65543502 | 2,546405061 | 0,59235472 | 4,298784116 | 1,72E-05 | 0,003393 | K04844 | ycjT | hypothetical glycosyl hydrolase [EC:3.2.1.-] |
| 110 | K08983 | 16,14686565 | 2,458305232 | 0,530923825 | 4,630240942 | 3,65E-06 | 0,001083 | K08983 | K08983 | putative membrane protein |
| 120 | K10193 | 69,8324474 | 2,428527134 | 0,579559803 | 4,190296015 | 2,79E-05 | 0,004244 | K10193 | togM | oligogalacturonide transport system permease protein |
| 121 | K10194 | 69,8324474 | 2,428527134 | 0,579559803 | 4,190296015 | 2,79E-05 | 0,004244 | K10194 | togN | oligogalacturonide transport system permease protein |
| 174 | K16188 | 4,962066914 | 2,392822237 | 0,45850964 | 5,218695587 | 1,80E-07 | 0,000137 | K16188 | ytpB | tetraprenyl-beta-curcumene synthase [EC:4.2.3.130] |
| 123 | K10242 | 5,136614165 | 2,279050508 | 0,471045782 | 4,838278136 | 1,31E-06 | 0,000454 | K10242 | cebG | cellobiose transport system permease protein |
| 175 | K16293 | 8,682702745 | 2,211907872 | 0,499631056 | 4,427082433 | 9,55E-06 | 0,002427 | K16293 | psrB | polysulfide reductase chain B |
| 176 | K16294 | 8,682702745 | 2,211907872 | 0,499631056 | 4,427082433 | 9,55E-06 | 0,002427 | K16294 | psrC | polysulfide reductase chain C |
| 137 | K12049 | 9,806906569 | 2,154504134 | 0,517776304 | 4,161071327 | 3,17E-05 | 0,004244 | K12049 | comB9 | ComB9 competence protein |
| 138 | K12050 | 9,806906569 | 2,154504134 | 0,517776304 | 4,161071327 | 3,17E-05 | 0,004244 | K12050 | comB8 | ComB8 competence protein |
| 139 | K12053 | 9,806906569 | 2,154504134 | 0,517776304 | 4,161071327 | 3,17E-05 | 0,004244 | K12053 | comB4 | ComB4 competence protein |
| 193 | K18989 | 4,875048945 | 2,152877185 | 0,504107058 | 4,270674552 | 1,95E-05 | 0,003466 | K18989 | vexF | multidrug efflux pump |
| 194 | K18990 | 4,875048945 | 2,152877185 | 0,504107058 | 4,270674552 | 1,95E-05 | 0,003466 | K18990 | vexE | membrane fusion protein, multidrug efflux system |
| 161 | K14977 | 16,14124 | 2,144935698 | 0,54418799 | 3,941534426 | 8,10E-05 | 0,007206 | K14977 | ylbA, UGHY | (S)-ureidoglycine aminohydrolase [EC:3.5.3.26] |
| 128 | K11003 | 8,701577317 | 2,138743778 | 0,488152381 | 4,381303587 | 1,18E-05 | 0,002736 | K11003 | hlyD, cyaD | hemolysin D |
| 1 | K00025 | 31,78196845 | 2,119437823 | 0,575925828 | 3,680053436 | 0,000233 | 0,013386 | K00025 | MDH1 | malate dehydrogenase [EC:1.1.1.37] |
| 131 | K11089 | 34,35336184 | 2,077096437 | 0,558571502 | 3,718586482 | 0,0002 | 0,012574 | K11089 | TROVE2, SSA2 | 60 kDa SS-A/Ro ribonucleoprotein |
| 119 | K09966 | 19,01179985 | 2,055340018 | 0,507722389 | 4,048157147 | 5,16E-05 | 0,005987 | K09966 | K09966 | uncharacterized protein |
| 172 | K16079 | 89,82991098 | 2,045243049 | 0,620416081 | 3,296566789 | 0,000979 | 0,032232 | K16079 | omp31 | outer membrane immunogenic protein |
| 106 | K08357 | 7,637433462 | 2,030998114 | 0,491189637 | 4,134855385 | 3,55E-05 | 0,004526 | K08357 | ttrA | tetrathionate reductase subunit A |
| 185 | K18284 | 12,08867295 | 2,017487274 | 0,506780348 | 3,980989561 | 6,86E-05 | 0,007111 | K18284 | K18284 | adenosylhomocysteine/aminodeoxyfutalosine nucleosidase [EC:3.2.2.9 3.2.2.30] |
| 134 | K11689 | 13,5816418 | 2,008497065 | 0,504035307 | 3,984834071 | 6,75E-05 | 0,007111 | K11689 | dctQ | C4-dicarboxylate transporter, DctQ subunit |
| 127 | K10943 | 12,74441997 | 1,994391783 | 0,491507474 | 4,057703874 | 4,96E-05 | 0,005875 | K10943 | flrC, fleR | two-component system, response regulator FlrC |
| 191 | K18923 | 12,6917146 | 1,984874784 | 0,495508192 | 4,005735562 | 6,18E-05 | 0,006872 | K18923 | stbD | antitoxin StbD |
| 12 | K00737 | 2,827035943 | 1,981009922 | 0,450085994 | 4,401403175 | 1,08E-05 | 0,002608 | K00737 | MGAT3 | beta-1,4-mannosyl-glycoprotein beta-1,4-N-acetylglucosaminyltransferase [EC:2.4.1.144] |
| 200 | K19156 | 3,631170663 | 1,977967257 | 0,458839826 | 4,310801163 | 1,63E-05 | 0,003338 | K19156 | prlF, sohA | antitoxin PrlF |
| 7 | K00469 | 4,580840652 | 1,953943014 | 0,448166903 | 4,359855671 | 1,30E-05 | 0,002893 | K00469 | MIOX | inositol oxygenase [EC:1.13.99.1] |
| 102 | K08093 | 51,30433437 | 1,949551002 | 0,547201638 | 3,562765288 | 0,000367 | 0,017732 | K08093 | hxlA | 3-hexulose-6-phosphate synthase [EC:4.1.2.43] |
| 104 | K08221 | 5,5610396 | 1,945132764 | 0,457875982 | 4,248165092 | 2,16E-05 | 0,003593 | K08221 | yitG, ymfD, yfmO | MFS transporter, ACDE family, multidrug resistance protein |
| 129 | K11004 | 7,951396652 | 1,935185376 | 0,495130417 | 3,908435653 | 9,29E-05 | 0,007994 | K11004 | hlyB, cyaB | ATP-binding cassette, subfamily B, bacterial HlyB/CyaB |
| 59 | K05299 | 9,310723499 | 1,931828797 | 0,490149266 | 3,941307134 | 8,10E-05 | 0,007206 | K05299 | fdhA | formate dehydrogenase (NADP+) alpha subunit [EC:1.17.1.10] |
| 51 | K03886 | 5,024800546 | 1,928582648 | 0,447219867 | 4,312381425 | 1,62E-05 | 0,003338 | K03886 | MQCRA, qcrA, bfcA, petC | menaquinol-cytochrome c reductase iron-sulfur subunit [EC:1.10.2.-] |
| 182 | K17250 | 7,73786034 | 1,919778399 | 0,493092652 | 3,893342136 | 9,89E-05 | 0,008267 | K17250 | pglI | GalNAc5-diNAcBac-PP-undecaprenol beta-1,3-glucosyltransferase [EC:2.4.1.293] |
| 115 | K09804 | 11,22212125 | 1,894611029 | 0,506541704 | 3,740286364 | 0,000184 | 0,012103 | K09804 | K09804 | uncharacterized protein |
| 66 | K05922 | 11,20700001 | 1,892358825 | 0,506760179 | 3,734229534 | 0,000188 | 0,012103 | K05922 | hydB | quinone-reactive Ni/Fe-hydrogenase large subunit [EC:1.12.5.1] |
| 67 | K05927 | 11,20700001 | 1,892358825 | 0,506760179 | 3,734229534 | 0,000188 | 0,012103 | K05927 | hydA | quinone-reactive Ni/Fe-hydrogenase small subunit [EC:1.12.5.1] |
| 101 | K08077 | 11,20700001 | 1,892358825 | 0,506760179 | 3,734229534 | 0,000188 | 0,012103 | K08077 | NUDT14 | UDP-sugar diphosphatase [EC:3.6.1.45] |
| 117 | K09944 | 11,20700001 | 1,892358825 | 0,506760179 | 3,734229534 | 0,000188 | 0,012103 | K09944 | K09944 | uncharacterized protein |
| 169 | K15910 | 11,18333393 | 1,889187593 | 0,509990049 | 3,70436168 | 0,000212 | 0,012814 | K15910 | pglE | UDP-N-acetylbacillosamine transaminase [EC:2.6.1.34] |
| 170 | K15912 | 11,18333393 | 1,889187593 | 0,509990049 | 3,70436168 | 0,000212 | 0,012814 | K15912 | pglF | UDP-N-acetyl-D-glucosamine 4,6-dehydratase [EC:4.2.1.135] |
| 171 | K15914 | 11,18333393 | 1,889187593 | 0,509990049 | 3,70436168 | 0,000212 | 0,012814 | K15914 | pglA | N,N'-diacetylbacillosaminyl-diphospho-undecaprenol alpha-1,3-N-acetylgalactosaminyltransferase [EC:2.4.1.290] |
| 199 | K19155 | 2,767232205 | 1,865796205 | 0,440988549 | 4,230940254 | 2,33E-05 | 0,003762 | K19155 | yhaV | toxin YhaV [EC:3.1.-.-] |
| 154 | K13819 | 11,93653268 | 1,846504277 | 0,492190061 | 3,751608213 | 0,000176 | 0,012103 | K13819 | K13819 | NifU-like protein |
| 153 | K13770 | 5,868962476 | 1,835813177 | 0,460564018 | 3,986010858 | 6,72E-05 | 0,007111 | K13770 | ysiA, fadR | TetR/AcrR family transcriptional regulator, fatty acid metabolism regulator protein |
| 184 | K18098 | 8,758992828 | 1,834019313 | 0,525057351 | 3,492988548 | 0,000478 | 0,021196 | K18098 | bjaR1, rpaR, rhiR | LuxR family transcriptional regulator, quorum-sensing system regulator BjaR1 |
| 2 | K00035 | 13,76072189 | 1,81200294 | 0,490024941 | 3,69777697 | 0,000217 | 0,012841 | K00035 | gal | D-galactose 1-dehydrogenase [EC:1.1.1.48] |
| 183 | K17251 | 12,60277516 | 1,783443437 | 0,518568874 | 3,439164068 | 0,000584 | 0,023584 | K17251 | pglB | undecaprenyl-diphosphooligosaccharide---protein glycotransferase [EC:2.4.99.19] |
| 168 | K15899 | 15,32767645 | 1,781591803 | 0,530742456 | 3,356791573 | 0,000789 | 0,028045 | K15899 | pseF | pseudaminic acid cytidylyltransferase [EC:2.7.7.81] |
| 109 | K08965 | 4,125145325 | 1,781371792 | 0,419163057 | 4,249830141 | 2,14E-05 | 0,003593 | K08965 | mtnW | 2,3-diketo-5-methylthiopentyl-1-phosphate enolase [EC:5.3.2.5] |
| 71 | K06311 | 2,560742926 | 1,766309278 | 0,427252478 | 4,134111254 | 3,56E-05 | 0,004526 | K06311 | yndE | spore germination protein |
| 181 | K17249 | 12,59798002 | 1,765818969 | 0,521993285 | 3,382838478 | 0,000717 | 0,026953 | K17249 | pglH | GalNAc-alpha-(1->4)-GalNAc-alpha-(1->3)-diNAcBac-PP-undecaprenol alpha-1,4-N-acetyl-D-galactosaminyltransferase [EC:2.4.1.292] |
| 32 | K02351 | 26,91949478 | 1,754128943 | 0,566602017 | 3,095874864 | 0,001962 | 0,048921 | K02351 | K02351 | putative membrane protein |
| 166 | K15896 | 10,18250683 | 1,751500634 | 0,505381224 | 3,465701828 | 0,000529 | 0,022042 | K15896 | pseH | UDP-4-amino-4,6-dideoxy-N-acetyl-beta-L-altrosamine N-acetyltransferase [EC:2.3.1.202] |
| 167 | K15897 | 10,18250683 | 1,751500634 | 0,505381224 | 3,465701828 | 0,000529 | 0,022042 | K15897 | pseG | UDP-2,4-diacetamido-2,4,6-trideoxy-beta-L-altropyranose hydrolase [EC:3.6.1.57] |
| 165 | K15895 | 10,20137776 | 1,733622095 | 0,505588638 | 3,428918225 | 0,000606 | 0,024308 | K15895 | pseC | UDP-4-amino-4,6-dideoxy-L-N-acetyl-beta-L-altrosamine transaminase [EC:2.6.1.92] |
| 180 | K16962 | 5,371861696 | 1,731493972 | 0,472885548 | 3,661549777 | 0,000251 | 0,014228 | K16962 | yxeN | putative amino-acid transport system permease protein |
| 81 | K06880 | 3,856767431 | 1,72603642 | 0,436511665 | 3,954158747 | 7,68E-05 | 0,007111 | K06880 | ereA_B | erythromycin esterase [EC:3.1.1.-] |
| 116 | K09860 | 12,29248213 | 1,721784642 | 0,489457504 | 3,517740823 | 0,000435 | 0,020056 | K09860 | K09860 | uncharacterized protein |
| 28 | K01959 | 14,20549258 | 1,716663361 | 0,509813554 | 3,367237586 | 0,000759 | 0,027555 | K01959 | pycA | pyruvate carboxylase subunit A [EC:6.4.1.1] |
| 36 | K02497 | 5,098299658 | 1,712179506 | 0,443370076 | 3,861738982 | 0,000113 | 0,00858 | K02497 | hemX | HemX protein |
| 34 | K02482 | 13,94669597 | 1,699053935 | 0,487567827 | 3,484754 | 0,000493 | 0,021541 | K02482 | K02482 | two-component system, NtrC family, sensor kinase [EC:2.7.13.3] |
| 41 | K02862 | 3,897893174 | 1,694625376 | 0,428545596 | 3,954364228 | 7,67E-05 | 0,007111 | K02862 | ctaG | putative membrane protein |
| 82 | K06882 | 11,65424257 | 1,689232412 | 0,514276535 | 3,284677207 | 0,001021 | 0,033012 | K06882 | K06882 | uncharacterized protein |
| 80 | K06425 | 5,473868119 | 1,660055663 | 0,453943445 | 3,656965823 | 0,000255 | 0,014333 | K06425 | sspH | small acid-soluble spore protein H (minor) |
| 108 | K08714 | 2,871738487 | 1,652394323 | 0,437052781 | 3,780766066 | 0,000156 | 0,011585 | K08714 | VGSC | voltage-gated sodium channel |
| 125 | K10549 | 2,950859823 | 1,644150444 | 0,441969993 | 3,720049934 | 0,000199 | 0,012574 | K10549 | alsB | D-allose transport system substrate-binding protein |
| 141 | K12278 | 5,071892718 | 1,626138073 | 0,45660509 | 3,561366508 | 0,000369 | 0,017732 | K12278 | mshG | MSHA biogenesis protein MshG |
| 113 | K09681 | 3,677699972 | 1,622012828 | 0,432061637 | 3,754123688 | 0,000174 | 0,012103 | K09681 | gltC | LysR family transcriptional regulator, transcription activator of glutamate synthase operon |
| 91 | K07283 | 13,30784104 | 1,621599974 | 0,500132489 | 3,242340798 | 0,001186 | 0,036349 | K07283 | ydiY | putative salt-induced outer membrane protein |
| 187 | K18554 | 5,770343785 | 1,619246703 | 0,491810585 | 3,292419381 | 0,000993 | 0,032511 | K18554 | cpt | chloramphenicol 3-O phosphotransferase [EC:2.7.1.-] |
| 162 | K15654 | 3,04696672 | 1,608915995 | 0,413866625 | 3,887522936 | 0,000101 | 0,008267 | K15654 | srfAA, lchAA | surfactin family lipopeptide synthetase A |
| 202 | K19687 | 4,013660104 | 1,584512788 | 0,477015038 | 3,32172502 | 0,000895 | 0,030595 | K19687 | vapB | antitoxin VapB |
| 148 | K13017 | 14,57700045 | 1,579388459 | 0,486238917 | 3,248173692 | 0,001161 | 0,036026 | K13017 | wbpE, wlbC | UDP-2-acetamido-2-deoxy-ribo-hexuluronate aminotransferase [EC:2.6.1.98] |
| 157 | K14166 | 8,827644694 | 1,577683113 | 0,506477459 | 3,115011507 | 0,001839 | 0,047178 | K14166 | ycnJ | copper transport protein |
| 158 | K14439 | 2,134287755 | 1,575111027 | 0,435827378 | 3,614070864 | 0,000301 | 0,015766 | K14439 | SMARCAD1 | SWI/SNF-related matrix-associated actin-dependent regulator of chromatin subfamily A containing DEAD/H box 1 [EC:3.6.4.12] |
| 147 | K12979 | 10,22210326 | 1,570652981 | 0,500425408 | 3,138635562 | 0,001697 | 0,045967 | K12979 | lpxO | beta-hydroxylase [EC:1.14.11.-] |
| 88 | K07215 | 90,70284349 | 1,531738008 | 0,464554928 | 3,29721614 | 0,000976 | 0,032232 | K07215 | pigA, hemO | heme oxygenase (biliverdin-IX-beta and delta-forming) [EC:1.14.99.58] |
| 136 | K11934 | 1513,121542 | 1,529557848 | 0,407493888 | 3,753572492 | 0,000174 | 0,012103 | K11934 | ompX | outer membrane protein X |
| 77 | K06380 | 2,913767979 | 1,518365001 | 0,422723069 | 3,591866909 | 0,000328 | 0,016218 | K06380 | spoIIB | stage II sporulation protein B |
| 73 | K06314 | 3,516175707 | 1,514684033 | 0,420337434 | 3,60349546 | 0,000314 | 0,015802 | K06314 | rsfA | prespore-specific regulator |
| 197 | K19137 | 7,732222177 | 1,50398771 | 0,465470807 | 3,231110707 | 0,001233 | 0,037378 | K19137 | csn2 | CRISPR-associated protein Csn2 |
| 76 | K06377 | 4,739487767 | 1,489096621 | 0,444528857 | 3,349831171 | 0,000809 | 0,028569 | K06377 | spo0M | sporulation-control protein |
| 133 | K11210 | 3,392405592 | 1,48835605 | 0,428091061 | 3,476727698 | 0,000508 | 0,021705 | K11210 | fosB | metallothiol transferase [EC:2.5.1.-] |
| 142 | K12287 | 2,106896094 | 1,487112463 | 0,410082818 | 3,626371056 | 0,000287 | 0,01553 | K12287 | mshQ | MSHA biogenesis protein MshQ |
| 189 | K18786 | 2,106896094 | 1,487112463 | 0,410082818 | 3,626371056 | 0,000287 | 0,01553 | K18786 | E2.4.1.321 | cellobionic acid phosphorylase [EC:2.4.1.321] |
| 114 | K09795 | 69,1998488 | 1,482504345 | 0,463906153 | 3,195698818 | 0,001395 | 0,040666 | K09795 | K09795 | uncharacterized protein |
| 100 | K07810 | 2,513054688 | 1,463514351 | 0,422148873 | 3,466820461 | 0,000527 | 0,022042 | K07810 | cusF | Cu(I)/Ag(I) efflux system periplasmic protein CusF |
| 97 | K07697 | 3,838979308 | 1,450752298 | 0,426956109 | 3,397895633 | 0,000679 | 0,025877 | K07697 | kinB | two-component system, sporulation sensor kinase B [EC:2.7.13.3] |
| 79 | K06417 | 3,308930027 | 1,448513476 | 0,416693432 | 3,476209042 | 0,000509 | 0,021705 | K06417 | spoVID | stage VI sporulation protein D |
| 55 | K03897 | 4,041821714 | 1,440816927 | 0,440977342 | 3,267326435 | 0,001086 | 0,034273 | K03897 | iucD | lysine N6-hydroxylase [EC:1.14.13.59] |
| 75 | K06349 | 3,351750467 | 1,432690584 | 0,416072932 | 3,443364073 | 0,000575 | 0,023398 | K06349 | kbaA | KinB signaling pathway activation protein |
| 140 | K12263 | 3,351750467 | 1,432690584 | 0,416072932 | 3,443364073 | 0,000575 | 0,023398 | K12263 | cccB | cytochrome c551 |
| 178 | K16856 | 3,132013424 | 1,427904485 | 0,456525951 | 3,127761918 | 0,001761 | 0,046436 | K16856 | ugl | ureidoglycolate lyase [EC:4.3.2.3] |
| 103 | K08174 | 2,778425659 | 1,420076478 | 0,414732788 | 3,424075742 | 0,000617 | 0,024419 | K08174 | glcP | MFS transporter, FHS family, glucose/mannose:H+ symporter |
| 70 | K06294 | 3,323567358 | 1,419033087 | 0,415207369 | 3,417649088 | 0,000632 | 0,024419 | K06294 | gerD | spore germination protein D |
| 72 | K06313 | 3,323567358 | 1,419033087 | 0,415207369 | 3,417649088 | 0,000632 | 0,024419 | K06313 | ypeB | spore germination protein |
| 96 | K07692 | 3,323567358 | 1,419033087 | 0,415207369 | 3,417649088 | 0,000632 | 0,024419 | K07692 | degU | two-component system, NarL family, response regulator DegU |
| 30 | K02239 | 3,30274416 | 1,407730985 | 0,417280044 | 3,373588089 | 0,000742 | 0,027299 | K02239 | comER | competence protein ComER |
| 74 | K06332 | 3,30274416 | 1,407730985 | 0,417280044 | 3,373588089 | 0,000742 | 0,027299 | K06332 | cotJA | spore coat protein JA |
| 179 | K16961 | 4,436090899 | 1,399379895 | 0,449975514 | 3,109902322 | 0,001871 | 0,047712 | K16961 | yxeM | putative amino-acid transport system substrate-binding protein |
| 152 | K13300 | 3,17614027 | 1,370852312 | 0,420063979 | 3,263436956 | 0,001101 | 0,034542 | K13300 | cccA | cytochrome c550 |
| 69 | K05997 | 2,479371215 | 1,359588611 | 0,402354389 | 3,379082346 | 0,000727 | 0,027133 | K05997 | sufA | Fe-S cluster assembly protein SufA |
| 35 | K02491 | 3,166567348 | 1,35308952 | 0,423343673 | 3,19619639 | 0,001393 | 0,040666 | K02491 | kinA | two-component system, sporulation sensor kinase A [EC:2.7.13.3] |
| 94 | K07445 | 1,969196412 | 1,348622777 | 0,385562366 | 3,497807085 | 0,000469 | 0,021031 | K07445 | K07445 | putative DNA methylase |
| 78 | K06401 | 3,160755765 | 1,333709526 | 0,41711878 | 3,197433416 | 0,001387 | 0,040666 | K06401 | spoIVFA | stage IV sporulation protein FA |
| 98 | K07717 | 2,507417915 | 1,315349268 | 0,410300803 | 3,205816944 | 0,001347 | 0,040141 | K07717 | ycbA, glnK | two-component system, sensor histidine kinase YcbA [EC:2.7.13.3] |
| 92 | K07339 | 485,6659613 | 1,315115876 | 0,378285223 | 3,476519289 | 0,000508 | 0,021705 | K07339 | hicA | mRNA interferase HicA [EC:3.1.-.-] |
| 52 | K03887 | 3,545173731 | 1,300101881 | 0,413239105 | 3,146125007 | 0,001654 | 0,045265 | K03887 | MQCRB, qcrB, bfcB, petB | menaquinol-cytochrome c reductase cytochrome b subunit |
| 53 | K03888 | 3,545173731 | 1,300101881 | 0,413239105 | 3,146125007 | 0,001654 | 0,045265 | K03888 | MQCRC, qcrC, bfcC, petD | menaquinol-cytochrome c reductase cytochrome b/c subunit |
| 95 | K07459 | 502,3070353 | 1,188249364 | 0,366326315 | 3,243690978 | 0,00118 | 0,036349 | K07459 | ybjD | putative ATP-dependent endonuclease of the OLD family |
| 13 | K00786 | 83,44248879 | 1,12363858 | 0,338012131 | 3,324255185 | 0,000887 | 0,030514 | K00786 | E2.4.-.- | glycosyltransferase [EC:2.4.-.-] |
| 18 | K01201 | 1250,414145 | 1,01410247 | 0,326197144 | 3,108863732 | 0,001878 | 0,047712 | K01201 | GBA, srfJ | glucosylceramidase [EC:3.2.1.45] |
| 86 | K07014 | 212,2886283 | -1,01644835 | 0,276218316 | -3,679873092 | 0,000233 | 0,013386 | K07014 | K07014 | uncharacterized protein |
| 60 | K05549 | 2,126451825 | -1,099270799 | 0,349077898 | -3,149070182 | 0,001638 | 0,045265 | K05549 | benA-xylX | benzoate/toluate 1,2-dioxygenase subunit alpha [EC:1.14.12.10 1.14.12.-] |
| 61 | K05550 | 2,126451825 | -1,099270799 | 0,349077898 | -3,149070182 | 0,001638 | 0,045265 | K05550 | benB-xylY | benzoate/toluate 1,2-dioxygenase subunit beta [EC:1.14.12.10 1.14.12.-] |
| 63 | K05783 | 2,126451825 | -1,099270799 | 0,349077898 | -3,149070182 | 0,001638 | 0,045265 | K05783 | benD-xylL | dihydroxycyclohexadiene carboxylate dehydrogenase [EC:1.3.1.25 1.3.1.-] |
| 40 | K02779 | 325,8205051 | -1,118307158 | 0,330451618 | -3,384178185 | 0,000714 | 0,026953 | K02779 | PTS-Glc-EIIC, ptsG | PTS system, glucose-specific IIC component |
| 4 | K00113 | 215,7979432 | -1,121548594 | 0,354394446 | -3,1646901 | 0,001552 | 0,044056 | K00113 | glpC | glycerol-3-phosphate dehydrogenase subunit C [EC:1.1.5.3] |
| 3 | K00112 | 215,7294584 | -1,122589594 | 0,355535877 | -3,157457988 | 0,001592 | 0,044688 | K00112 | glpB | glycerol-3-phosphate dehydrogenase subunit B [EC:1.1.5.3] |
| 50 | K03762 | 99,934551 | -1,146417137 | 0,367560625 | -3,118987883 | 0,001815 | 0,047089 | K03762 | proP | MFS transporter, MHS family, proline/betaine transporter |
| 9 | K00598 | 116,6571093 | -1,174020636 | 0,353804905 | -3,318271224 | 0,000906 | 0,030779 | K00598 | tam | trans-aconitate 2-methyltransferase [EC:2.1.1.144] |
| 16 | K01067 | 2,525768898 | -1,22935911 | 0,396865512 | -3,097671811 | 0,00195 | 0,048917 | K01067 | E3.1.2.1, ACH1 | acetyl-CoA hydrolase [EC:3.1.2.1] |
| 38 | K02552 | 145,5602246 | -1,242629468 | 0,398485989 | -3,118376809 | 0,001819 | 0,047089 | K02552 | menF | menaquinone-specific isochorismate synthase [EC:5.4.4.2] |
| 37 | K02549 | 165,2436738 | -1,273977978 | 0,377919306 | -3,371031745 | 0,000749 | 0,027365 | K02549 | menC | O-succinylbenzoate synthase [EC:4.2.1.113] |
| 195 | K18996 | 2,104072832 | -1,28443838 | 0,388530658 | -3,30588682 | 0,000947 | 0,031767 | K18996 | repC | replication initiation protein RepC |
| 21 | K01535 | 2,05405067 | -1,288085327 | 0,357226042 | -3,605799064 | 0,000311 | 0,015802 | K01535 | PMA1, PMA2 | H+-transporting ATPase [EC:3.6.3.6] |
| 68 | K05962 | 2,691389906 | -1,288426808 | 0,404609869 | -3,184368222 | 0,001451 | 0,041388 | K05962 | E2.7.13.1 | protein-histidine pros-kinase [EC:2.7.13.1] |
| 26 | K01792 | 205,2536432 | -1,304749231 | 0,336536049 | -3,876996934 | 0,000106 | 0,008306 | K01792 | E5.1.3.15 | glucose-6-phosphate 1-epimerase [EC:5.1.3.15] |
| 23 | K01576 | 3,234431693 | -1,31341659 | 0,420432887 | -3,123962541 | 0,001784 | 0,046664 | K01576 | mdlC | benzoylformate decarboxylase [EC:4.1.1.7] |
| 118 | K09964 | 5,869809893 | -1,329552283 | 0,41253367 | -3,222893983 | 0,001269 | 0,038035 | K09964 | K09964 | uncharacterized protein |
| 159 | K14660 | 3,061867633 | -1,33189182 | 0,376965202 | -3,533195674 | 0,000411 | 0,019214 | K14660 | nodE | nodulation protein E [EC:2.3.1.-] |
| 20 | K01485 | 243,7627168 | -1,342695208 | 0,362677496 | -3,702174036 | 0,000214 | 0,012814 | K01485 | codA | cytosine deaminase [EC:3.5.4.1] |
| 124 | K10533 | 5,583449138 | -1,369798866 | 0,4280706 | -3,199936804 | 0,001375 | 0,040666 | K10533 | E3.3.2.8 | limonene-1,2-epoxide hydrolase [EC:3.3.2.8] |
| 25 | K01768 | 150,220855 | -1,373719103 | 0,402449922 | -3,413391404 | 0,000642 | 0,024625 | K01768 | E4.6.1.1 | adenylate cyclase [EC:4.6.1.1] |
| 48 | K03668 | 18,891512 | -1,380122731 | 0,423259302 | -3,260702661 | 0,001111 | 0,034673 | K03668 | hslJ | heat shock protein HslJ |
| 144 | K12537 | 3,782865171 | -1,3839485 | 0,441913175 | -3,131720387 | 0,001738 | 0,046374 | K12537 | hasE, prtE | membrane fusion protein, protease secretion system |
| 49 | K03757 | 10,87850375 | -1,414752286 | 0,450413071 | -3,141010724 | 0,001684 | 0,045828 | K03757 | cadB | cadaverine:lysine antiporter |
| 145 | K12538 | 3,751024256 | -1,416915433 | 0,443882261 | -3,19209745 | 0,001412 | 0,040742 | K12538 | hasF, prtF | outer membrane protein, protease secretion system |
| 201 | K19504 | 3,384707184 | -1,434668499 | 0,397889538 | -3,605695451 | 0,000311 | 0,015802 | K19504 | gfrE | glucoselysine-6-phosphate deglycase |
| 150 | K13059 | 59,16666648 | -1,448129173 | 0,442444601 | -3,273018068 | 0,001064 | 0,033992 | K13059 | nahK, lnpB | N-acetylhexosamine 1-kinase [EC:2.7.1.162] |
| 84 | K06992 | 8,341422406 | -1,451135525 | 0,448020179 | -3,238995907 | 0,0012 | 0,036568 | K06992 | K06992 | uncharacterized protein |
| 155 | K13875 | 4,037570134 | -1,473591768 | 0,440923194 | -3,342059994 | 0,000832 | 0,028997 | K13875 | K13875, araC | L-arabonate dehydrase [EC:4.2.1.25] |
| 62 | K05596 | 48,15316069 | -1,476477613 | 0,439813628 | -3,357052895 | 0,000788 | 0,028045 | K05596 | iciA | LysR family transcriptional regulator, chromosome initiation inhibitor |
| 105 | K08225 | 13,5478013 | -1,485809508 | 0,454348293 | -3,270199386 | 0,001075 | 0,034129 | K08225 | entS | MFS transporter, ENTS family, enterobactin (siderophore) exporter |
| 112 | K09456 | 46,11277891 | -1,497981381 | 0,385568461 | -3,885124256 | 0,000102 | 0,008267 | K09456 | aidB | putative acyl-CoA dehydrogenase |
| 29 | K02182 | 30,83776336 | -1,515303315 | 0,469613716 | -3,226701574 | 0,001252 | 0,037745 | K02182 | caiC | carnitine-CoA ligase [EC:6.2.1.48] |
| 47 | K03489 | 25,62534285 | -1,524280397 | 0,433375919 | -3,517224493 | 0,000436 | 0,020056 | K03489 | yydK | GntR family transcriptional regulator, transcriptional regulator of bglA |
| 42 | K03287 | 5,04686937 | -1,52879863 | 0,407534725 | -3,751333413 | 0,000176 | 0,012103 | K03287 | TC.OMF | outer membrane factor, OMF family |
| 45 | K03433 | 2,637863797 | -1,530534755 | 0,38652865 | -3,959692911 | 7,50E-05 | 0,007111 | K03433 | psmB, prcB | proteasome beta subunit [EC:3.4.25.1] |
| 163 | K15781 | 2,73125805 | -1,554979711 | 0,399965948 | -3,88778024 | 0,000101 | 0,008267 | K15781 | serB-plsC | putative phosphoserine phosphatase / 1-acylglycerol-3-phosphate O-acyltransferase [EC:3.1.3.3 2.3.1.51] |
| 43 | K03333 | 6,892847806 | -1,571680025 | 0,477767888 | -3,289630936 | 0,001003 | 0,032634 | K03333 | choD | cholesterol oxidase [EC:1.1.3.6] |
| 64 | K05793 | 8,528153943 | -1,614189691 | 0,468075434 | -3,448567417 | 0,000564 | 0,023307 | K05793 | terB | tellurite resistance protein TerB |
| 135 | K11750 | 13,32189753 | -1,631819048 | 0,452555377 | -3,605788661 | 0,000311 | 0,015802 | K11750 | frsA | esterase FrsA [EC:3.1.-.-] |
| 56 | K04091 | 72,75728522 | -1,635184128 | 0,494465897 | -3,306970486 | 0,000943 | 0,031767 | K04091 | ssuD | alkanesulfonate monooxygenase [EC:1.14.14.5] |
| 14 | K00897 | 22,00808169 | -1,667650539 | 0,504901839 | -3,302920313 | 0,000957 | 0,031904 | K00897 | aphA | kanamycin kinase [EC:2.7.1.95] |
| 99 | K07771 | 18,2204359 | -1,670403599 | 0,47684844 | -3,503007369 | 0,00046 | 0,020799 | K07771 | basR | two-component system, OmpR family, response regulator BasR |
| 192 | K18988 | 12,75202258 | -1,676300424 | 0,453546506 | -3,695983549 | 0,000219 | 0,012841 | K18988 | ampH | serine-type D-Ala-D-Ala carboxypeptidase/endopeptidase [EC:3.4.16.4 3.4.21.-] |
| 5 | K00355 | 127,9774464 | -1,682392224 | 0,475106286 | -3,541086009 | 0,000398 | 0,018813 | K00355 | NQO1 | NAD(P)H dehydrogenase (quinone) [EC:1.6.5.2] |
| 17 | K01121 | 4,581447734 | -1,703236143 | 0,414381805 | -4,110306295 | 3,95E-05 | 0,004874 | K01121 | CNP | 2',3'-cyclic-nucleotide 3'-phosphodiesterase [EC:3.1.4.37] |
| 24 | K01608 | 20,46404835 | -1,744900607 | 0,466938049 | -3,7368996 | 0,000186 | 0,012103 | K01608 | gcl | tartronate-semialdehyde synthase [EC:4.1.1.47] |
| 39 | K02616 | 16,77718706 | -1,74579651 | 0,546924592 | -3,192024157 | 0,001413 | 0,040742 | K02616 | paaX | phenylacetic acid degradation operon negative regulatory protein |
| 132 | K11159 | 22,60558367 | -1,757631559 | 0,522673465 | -3,36277174 | 0,000772 | 0,027816 | K11159 | K11159 | carotenoid cleavage dioxygenase |
| 33 | K02480 | 9,834261929 | -1,759294616 | 0,495326212 | -3,551789861 | 0,000383 | 0,018226 | K02480 | K02480 | two-component system, NarL family, sensor kinase [EC:2.7.13.3] |
| 8 | K00588 | 24,56707962 | -1,772292038 | 0,529997118 | -3,343965425 | 0,000826 | 0,028988 | K00588 | E2.1.1.104 | caffeoyl-CoA O-methyltransferase [EC:2.1.1.104] |
| 111 | K09000 | 6,331919866 | -1,781415269 | 0,492728391 | -3,615410239 | 0,0003 | 0,015766 | K09000 | cmr4 | CRISPR-associated protein Cmr4 |
| 196 | K19076 | 6,331919866 | -1,781415269 | 0,492728391 | -3,615410239 | 0,0003 | 0,015766 | K19076 | cmr2, cas10 | CRISPR-associated protein Cmr2 |
| 146 | K12954 | 29,44765244 | -1,782594177 | 0,51058568 | -3,491273347 | 0,000481 | 0,021196 | K12954 | ctpG | cation-transporting P-type ATPase G [EC:3.6.3.-] |
| 198 | K19141 | 6,319945062 | -1,789886937 | 0,493667454 | -3,625693621 | 0,000288 | 0,01553 | K19141 | cmr5 | CRISPR-associated protein Cmr5 |
| 89 | K07222 | 10,76914637 | -1,799192173 | 0,495387773 | -3,631886515 | 0,000281 | 0,01553 | K07222 | K07222 | putative flavoprotein involved in K+ transport |
| 54 | K03891 | 8,620380104 | -1,809982603 | 0,506715244 | -3,571991618 | 0,000354 | 0,01734 | K03891 | qcrB | ubiquinol-cytochrome c reductase cytochrome b subunit |
| 15 | K00966 | 10,71587657 | -1,810508524 | 0,467020302 | -3,876723379 | 0,000106 | 0,008306 | K00966 | GMPP | mannose-1-phosphate guanylyltransferase [EC:2.7.7.13] |
| 143 | K12448 | 6,066270856 | -1,861713868 | 0,468242036 | -3,975964832 | 7,01E-05 | 0,007111 | K12448 | UXE, uxe | UDP-arabinose 4-epimerase [EC:5.1.3.5] |
| 19 | K01269 | 9,566581303 | -1,865464093 | 0,492522445 | -3,787571738 | 0,000152 | 0,011431 | K01269 | E3.4.11.- | aminopeptidase [EC:3.4.11.-] |
| 44 | K03381 | 21,85419725 | -1,867323398 | 0,559349236 | -3,338385534 | 0,000843 | 0,029192 | K03381 | catA | catechol 1,2-dioxygenase [EC:1.13.11.1] |
| 130 | K11016 | 2,242072315 | -1,877899561 | 0,362449098 | -5,181140111 | 2,21E-07 | 0,000147 | K11016 | shlA, hhdA, hpmA | hemolysin |
| 57 | K04093 | 7,767431578 | -1,893207026 | 0,477279004 | -3,966667315 | 7,29E-05 | 0,007111 | K04093 | pheA1 | chorismate mutase [EC:5.4.99.5] |
| 85 | K06995 | 31,1433644 | -1,893226848 | 0,553442769 | -3,420817751 | 0,000624 | 0,024419 | K06995 | K06995 | uncharacterized protein |
| 22 | K01563 | 21,29457808 | -1,913072654 | 0,459871051 | -4,160019748 | 3,18E-05 | 0,004244 | K01563 | dhaA | haloalkane dehalogenase [EC:3.8.1.5] |
| 31 | K02336 | 18,84481058 | -1,928887747 | 0,46345881 | -4,161939977 | 3,16E-05 | 0,004244 | K02336 | polB | DNA polymerase II [EC:2.7.7.7] |
| 6 | K00452 | 19,75984593 | -1,935196297 | 0,49407397 | -3,91681492 | 8,97E-05 | 0,007847 | K00452 | HAAO | 3-hydroxyanthranilate 3,4-dioxygenase [EC:1.13.11.6] |
| 93 | K07396 | 19,42806873 | -1,987198056 | 0,501304038 | -3,964057547 | 7,37E-05 | 0,007111 | K07396 | K07396 | putative protein-disulfide isomerase |
| 90 | K07241 | 8,074776054 | -1,988282145 | 0,463882685 | -4,286174525 | 1,82E-05 | 0,003463 | K07241 | nixA | high-affinity nickel-transport protein |
| 87 | K07161 | 25,5102029 | -2,036982516 | 0,526402275 | -3,869630915 | 0,000109 | 0,008428 | K07161 | K07161 | uncharacterized protein |
| 190 | K18901 | 16,9492104 | -2,073580829 | 0,504970006 | -4,106344546 | 4,02E-05 | 0,004874 | K18901 | bpeE | membrane fusion protein, multidrug efflux system |
| 173 | K16135 | 17,86880133 | -2,088120596 | 0,46853457 | -4,456705498 | 8,32E-06 | 0,002337 | K16135 | dmlR | LysR family transcriptional regulator, transcriptional activator for dmlA |
| 186 | K18351 | 15,55906056 | -2,115097865 | 0,524059218 | -4,035990191 | 5,44E-05 | 0,006172 | K18351 | vanSAc | two-component system, OmpR family, sensor histidine kinase VanS [EC:2.7.13.3] |
| 188 | K18704 | 36,23209014 | -2,117093487 | 0,588711289 | -3,596148957 | 0,000323 | 0,016103 | K18704 | tarL | CDP-ribitol ribitolphosphotransferase / teichoic acid ribitol-phosphate polymerase [EC:2.7.8.14 2.7.8.47] |
| 65 | K05898 | 4,534736559 | -2,219115802 | 0,45565559 | -4,870160376 | 1,12E-06 | 0,000454 | K05898 | kstD | 3-oxosteroid 1-dehydrogenase [EC:1.3.99.4] |
| 11 | K00663 | 19,93564661 | -2,227846077 | 0,563639851 | -3,952605683 | 7,73E-05 | 0,007111 | K00663 | aacA | aminoglycoside 6'-N-acetyltransferase [EC:2.3.1.82] |
| 160 | K14743 | 6,294210041 | -2,315612029 | 0,479305359 | -4,831183266 | 1,36E-06 | 0,000454 | K14743 | mycP | membrane-anchored mycosin MYCP [EC:3.4.21.-] |
| 151 | K13060 | 6,417984955 | -2,322076274 | 0,473522939 | -4,903830597 | 9,40E-07 | 0,000418 | K13060 | lasI, luxI | acyl homoserine lactone synthase [EC:2.3.1.184] |
| 27 | K01856 | 8,891866196 | -2,34436772 | 0,491970322 | -4,765262487 | 1,89E-06 | 0,000592 | K01856 | catB | muconate cycloisomerase [EC:5.5.1.1] |
| 149 | K13028 | 5,985929893 | -2,373842018 | 0,482717157 | -4,917666559 | 8,76E-07 | 0,000418 | K13028 | oxdA | aldoxime dehydratase [EC:4.99.1.5] |
| 83 | K06990 | 15,88545626 | -2,474350102 | 0,488349991 | -5,066755704 | 4,05E-07 | 0,000216 | K06990 | MEMO1 | MEMO1 family protein |
| 164 | K15792 | 8,457001954 | -2,712509397 | 0,492710546 | -5,505279755 | 3,69E-08 | 3,28E-05 | K15792 | murEF | murE/murF fusion protein [EC:6.3.2.13 6.3.2.10] |
| 177 | K16841 | 19,71762614 | -2,917115532 | 0,511386348 | -5,704328138 | 1,17E-08 | 1,25E-05 | K16841 | hpxA | allantoin racemase [EC:5.1.99.3] |
| 46 | K03464 | 15,50540673 | -3,206453651 | 0,498526336 | -6,431864113 | 1,26E-10 | 3,36E-07 | K03464 | catC | muconolactone D-isomerase [EC:5.3.3.4] |

Table 1 suppl. DESeq2-identified differentially abundant PICRUSt2-predicted KO functions encoded by

genomes of bacteria thriving in fecal samples.
