# Supplementary material for: The oral cavity and intestinal microbiome in children with functional constipation
Source: Sci Rep. 2024 Apr 9;14:8283. doi: 10.1038/s41598-024-58642-2 (PMC11004141; doi:10.1038/s41598-024-58642-2)
Supplement: Supplementary file 2 — Supplementary Table 2. [file 41598_2024_58642_MOESM2_ESM.docx]

|  | Row.names | baseMean | log2FoldChange | lfcSE | stat | pvalue | padj | fun | desc |
| --- | --- | --- | --- | --- | --- | --- | --- | --- | --- |
| 2 | PWY-5651 | 24,08073739 | -1,83627 | 0,503319179 | -3,648316615 | 0,000263964 | 0,044609953 | PWY-5651 | L-tryptophan degradation to 2-amino-3-carboxymuconate semialdehyde |
| 1 | CATECHOL-ORTHO-CLEAVAGE-PWY | 16,53182813 | -2,02534 | 0,554971708 | -3,64945204 | 0,0002628 | 0,044609953 | CATECHOL-ORTHO-CLEAVAGE-PWY | catechol degradation to &beta;-ketoadipate |

Table 2 suppl. DESeq2-identified differentially abundant PICRUSt2-predicted pathways encoded by

genomes of bacteria thriving in fecal samples.
