# Supplementary material for: The oral cavity and intestinal microbiome in children with functional constipation
Source: Sci Rep. 2024 Apr 9;14:8283. doi: 10.1038/s41598-024-58642-2 (PMC11004141; doi:10.1038/s41598-024-58642-2)
Supplement: Supplementary file 3 — Supplementary Table 3. [file 41598_2024_58642_MOESM3_ESM.docx]

|  | Row.names | baseMean | log2FoldChange | lfcSE | stat | pvalue | padj | fun | desc |  |
| --- | --- | --- | --- | --- | --- | --- | --- | --- | --- | --- |
| 113 | K16849 | 12,80238571 | 3,354587739 | 0,54969772 | 6,102604428 | 1,04E-09 | 1,96E-06 | K16849 | uxaA1 | altronate dehydratase small subunit [EC:4.2.1.7] |
| 54 | K08226 | 11,32477007 | 3,005119649 | 0,549826683 | 5,465576232 | 4,61E-08 | 2,89E-05 | K08226 | pucC | MFS transporter, BCD family, chlorophyll transporter |
| 39 | K05523 | 7,651962087 | 2,890182471 | 0,5209913 | 5,547467819 | 2,90E-08 | 2,33E-05 | K05523 | hchA | D-lactate dehydratase / protein deglycase [EC:4.2.1.130 3.5.1.124] |
| 78 | K12061 | 8,557794004 | 2,705422257 | 0,50419063 | 5,365871743 | 8,06E-08 | 4,53E-05 | K12061 | traW | conjugal transfer pilus assembly protein TraW |
| 79 | K12064 | 10,6872222 | 2,523655447 | 0,529675051 | 4,764535237 | 1,89E-06 | 0,000561 | K12064 | traV | conjugal transfer pilus assembly protein TraV |
| 138 | K19701 | 5,912739053 | 2,448642384 | 0,527922335 | 4,638262526 | 3,51E-06 | 0,000942 | K19701 | ywaD | aminopeptidase YwaD [EC:3.4.11.6 3.4.11.10] |
| 76 | K12056 | 11,13307595 | 2,435600998 | 0,52215748 | 4,664495085 | 3,09E-06 | 0,000871 | K12056 | traG | conjugal transfer mating pair stabilization protein TraG |
| 120 | K18209 | 4,673938333 | 2,369907835 | 0,491039679 | 4,826306175 | 1,39E-06 | 0,00053 | K18209 | tfrA | fumarate reductase (CoM/CoB) subunit A [EC:1.3.4.1] |
| 126 | K19051 | 9,207385071 | 2,293286706 | 0,451134844 | 5,083373046 | 3,71E-07 | 0,00019 | K19051 | hepB | heparin/heparan-sulfate lyase [EC:4.2.2.7 4.2.2.8] |
| 87 | K13069 | 12,78070169 | 2,275197714 | 0,475295703 | 4,786909919 | 1,69E-06 | 0,00053 | K13069 | E2.7.7.65 | diguanylate cyclase [EC:2.7.7.65] |
| 57 | K08720 | 4,031944472 | 2,254092846 | 0,487561545 | 4,623196532 | 3,78E-06 | 0,000967 | K08720 | ompU | outer membrane protein OmpU |
| 77 | K12059 | 5,394184229 | 2,226672407 | 0,484968232 | 4,591377873 | 4,40E-06 | 0,001058 | K12059 | trbC | conjugal transfer pilus assembly protein TrbC |
| 82 | K12068 | 8,63849892 | 2,181742693 | 0,509573747 | 4,281505287 | 1,86E-05 | 0,001972 | K12068 | traL | conjugal transfer pilus assembly protein TraL |
| 81 | K12066 | 6,12189998 | 2,172754681 | 0,486710435 | 4,46416293 | 8,04E-06 | 0,001321 | K12066 | traK | conjugal transfer pilus assembly protein TraK |
| 40 | K05548 | 13,59551885 | 2,164512405 | 0,527440882 | 4,103800975 | 4,06E-05 | 0,00375 | K05548 | benK | MFS transporter, AAHS family, benzoate transport protein |
| 20 | K02597 | 4,096179329 | 2,153856641 | 0,470065924 | 4,582031008 | 4,60E-06 | 0,001058 | K02597 | nifZ | nitrogen fixation protein NifZ |
| 23 | K03403 | 5,150018897 | 2,129101102 | 0,481095896 | 4,42552331 | 9,62E-06 | 0,001321 | K03403 | chlH, bchH | magnesium chelatase subunit H [EC:6.6.1.1] |
| 24 | K03428 | 5,150018897 | 2,129101102 | 0,481095896 | 4,42552331 | 9,62E-06 | 0,001321 | K03428 | bchM, chlM | magnesium-protoporphyrin O-methyltransferase [EC:2.1.1.11] |
| 31 | K04038 | 5,150018897 | 2,129101102 | 0,481095896 | 4,42552331 | 9,62E-06 | 0,001321 | K04038 | chlN | light-independent protochlorophyllide reductase subunit N [EC:1.3.7.7] |
| 73 | K10960 | 5,150018897 | 2,129101102 | 0,481095896 | 4,42552331 | 9,62E-06 | 0,001321 | K10960 | chlP, bchP | geranylgeranyl diphosphate/geranylgeranyl-bacteriochlorophyllide a reductase [EC:1.3.1.83 1.3.1.111] |
| 53 | K07807 | 5,880852468 | 2,114283226 | 0,472850124 | 4,471360206 | 7,77E-06 | 0,001321 | K07807 | K07807 | uncharacterized protein |
| 117 | K17465 | 4,921229655 | 2,102666071 | 0,467362194 | 4,499007621 | 6,83E-06 | 0,001321 | K17465 | PTS-Dga-EIIB, dgaB | PTS system, D-glucosaminate-specific IIB component [EC:2.7.1.203] |
| 133 | K19181 | 6,216852915 | 2,098754815 | 0,482425735 | 4,350420515 | 1,36E-05 | 0,001633 | K19181 | afr | 1,5-anhydro-D-fructose reductase (1,5-anhydro-D-mannitol-forming) [EC:1.1.1.292] |
| 59 | K08997 | 4,420035957 | 2,077867591 | 0,433297725 | 4,795473113 | 1,62E-06 | 0,00053 | K08997 | ydiU | uncharacterized protein |
| 94 | K14159 | 4,413298666 | 2,063920151 | 0,47039354 | 4,387645612 | 1,15E-05 | 0,001536 | K14159 | rnhA-dnaQ | ribonuclease HI / DNA polymerase III subunit epsilon [EC:3.1.26.4 2.7.7.7] |
| 127 | K19056 | 35,44079377 | 2,025286832 | 0,542896729 | 3,730519494 | 0,000191 | 0,013117 | K19056 | tetD | AraC family transcriptional regulator, transposon Tn10 TetD protein |
| 125 | K19047 | 35,29981409 | 2,018745157 | 0,543980339 | 3,711062721 | 0,000206 | 0,013545 | K19047 | tetC | TetR/AcrR family transcriptional regulator, repressor of tetCD |
| 43 | K05984 | 4,989136034 | 1,991691104 | 0,464951122 | 4,283656952 | 1,84E-05 | 0,001972 | K05984 | cho | excinuclease Cho [EC:3.1.25.-] |
| 84 | K12243 | 7,019842142 | 1,932580358 | 0,496564114 | 3,891904999 | 9,95E-05 | 0,007367 | K12243 | pchR | AraC family transcriptional regulator, transcriptional activator of the genes for pyochelin and ferripyochelin receptors |
| 80 | K12065 | 11,44537947 | 1,931637898 | 0,528869971 | 3,652387173 | 0,00026 | 0,015731 | K12065 | traB | conjugal transfer pilus assembly protein TraB |
| 2 | K00132 | 3,311087127 | 1,927840854 | 0,477707506 | 4,035609305 | 5,45E-05 | 0,00479 | K00132 | E1.2.1.10 | acetaldehyde dehydrogenase (acetylating) [EC:1.2.1.10] |
| 69 | K10565 | 6,078215211 | 1,922429376 | 0,478370657 | 4,018702544 | 5,85E-05 | 0,004943 | K10565 | motD | chemotaxis protein MotD |
| 36 | K05338 | 8,270246171 | 1,895557197 | 0,478387006 | 3,962392735 | 7,42E-05 | 0,006053 | K05338 | lrgA | holin-like protein |
| 122 | K18476 | 36,41989495 | 1,853281077 | 0,532202937 | 3,482282694 | 0,000497 | 0,022388 | K18476 | tetR | TetR/AcrR family transcriptional regulator, tetracycline repressor protein |
| 121 | K18243 | 5,192264631 | 1,838768871 | 0,471949522 | 3,896113429 | 9,77E-05 | 0,007367 | K18243 | nagH | salicylate 5-hydroxylase small subunit [EC:1.14.13.172] |
| 35 | K04775 | 3,614852198 | 1,820890724 | 0,419516003 | 4,340455932 | 1,42E-05 | 0,001633 | K04775 | ydgD | protease YdgD [EC:3.4.21.-] |
| 9 | K00801 | 7,368905736 | 1,812232276 | 0,488421997 | 3,710382183 | 0,000207 | 0,013545 | K00801 | FDFT1 | farnesyl-diphosphate farnesyltransferase [EC:2.5.1.21] |
| 28 | K03897 | 9,012742057 | 1,782084358 | 0,455914386 | 3,908813613 | 9,28E-05 | 0,007152 | K03897 | iucD | lysine N6-hydroxylase [EC:1.14.13.59] |
| 34 | K04110 | 8,477168897 | 1,781813115 | 0,472047734 | 3,774646049 | 0,00016 | 0,011444 | K04110 | badA | benzoate-CoA ligase [EC:6.2.1.25] |
| 30 | K04035 | 4,296091024 | 1,764627779 | 0,467566047 | 3,774071684 | 0,000161 | 0,011444 | K04035 | E1.14.13.81, acsF, chlE | magnesium-protoporphyrin IX monomethyl ester (oxidative) cyclase [EC:1.14.13.81] |
| 102 | K15654 | 7,813481333 | 1,760066809 | 0,48226885 | 3,649555238 | 0,000263 | 0,015731 | K15654 | srfAA, lchAA | surfactin family lipopeptide synthetase A |
| 64 | K09470 | 2,906313627 | 1,756494021 | 0,431199797 | 4,073503823 | 4,63E-05 | 0,004138 | K09470 | puuA | gamma-glutamylputrescine synthase [EC:6.3.1.11] |
| 3 | K00298 | 11,35195864 | 1,741213846 | 0,52217275 | 3,334555178 | 0,000854 | 0,033011 | K00298 | ceo | N5-(carboxyethyl)ornithine synthase [EC:1.5.1.24] |
| 37 | K05339 | 6,431483718 | 1,705374844 | 0,470436533 | 3,625090153 | 0,000289 | 0,016112 | K05339 | lrgB | holin-like protein LrgB |
| 58 | K08927 | 3,945133398 | 1,675077345 | 0,462494481 | 3,621832074 | 0,000293 | 0,016143 | K08927 | pufB | light-harvesting complex 1 beta chain |
| 65 | K09966 | 20,74832479 | 1,668508772 | 0,477582071 | 3,493658733 | 0,000476 | 0,021804 | K09966 | K09966 | uncharacterized protein |
| 137 | K19592 | 4,623366941 | 1,629462688 | 0,461068342 | 3,534102299 | 0,000409 | 0,020382 | K19592 | golS | MerR family transcriptional regulator, gold-responsive activator of gol and ges genes |
| 42 | K05817 | 2,723045102 | 1,611295472 | 0,43757218 | 3,682353556 | 0,000231 | 0,014639 | K05817 | hcaR | LysR family transcriptional regulator, hca operon transcriptional activator |
| 139 | K19731 | 5,858410875 | 1,608552786 | 0,473028911 | 3,400538002 | 0,000673 | 0,028679 | K19731 | cciR | LuxR family transcriptional regulator, quorum-sensing system regulator CciR |
| 90 | K13794 | 8,09577458 | 1,60325283 | 0,475253034 | 3,373472059 | 0,000742 | 0,030277 | K13794 | tcuR | LysR family transcriptional regulator, regulatory protein for tcuABC |
| 99 | K15534 | 2,801631956 | 1,595750578 | 0,433397701 | 3,681954416 | 0,000231 | 0,014639 | K15534 | K15534 | beta-D-galactosyl-(1->4)-L-rhamnose phosphorylase [EC:2.4.1.247] |
| 1 | K00090 | 26,92296916 | 1,581987462 | 0,425385416 | 3,718950865 | 0,0002 | 0,013545 | K00090 | ghrB | glyoxylate/hydroxypyruvate/2-ketogluconate reductase [EC:1.1.1.79 1.1.1.81 1.1.1.215] |
| 100 | K15551 | 11,83295245 | 1,570225659 | 0,468850584 | 3,349096095 | 0,000811 | 0,032833 | K15551 | tauA | taurine transport system substrate-binding protein |
| 111 | K16347 | 4,143109516 | 1,559592626 | 0,427308064 | 3,649808551 | 0,000262 | 0,015731 | K16347 | ecnA | entericidin A |
| 61 | K09024 | 15,37710946 | 1,544065468 | 0,424540942 | 3,637023699 | 0,000276 | 0,016112 | K09024 | rutF | flavin reductase [EC:1.5.1.-] |
| 71 | K10831 | 11,24374575 | 1,541663801 | 0,466593197 | 3,304085464 | 0,000953 | 0,034793 | K10831 | tauB | taurine transport system ATP-binding protein [EC:3.6.3.36] |
| 66 | K10036 | 7,290621294 | 1,523698869 | 0,460729167 | 3,307146541 | 0,000943 | 0,034676 | K10036 | glnH | glutamine transport system substrate-binding protein |
| 32 | K04103 | 4,5011131 | 1,521984459 | 0,439412973 | 3,463676657 | 0,000533 | 0,023724 | K04103 | ipdC | indolepyruvate decarboxylase [EC:4.1.1.74] |
| 46 | K07063 | 2,694937368 | 1,498970117 | 0,436789929 | 3,431787269 | 0,0006 | 0,025963 | K07063 | K07063 | uncharacterized protein |
| 136 | K19430 | 2,560145027 | 1,495807368 | 0,412267779 | 3,628242235 | 0,000285 | 0,016112 | K19430 | epsN | pyridoxal phosphate-dependent aminotransferase EpsN [EC:2.6.1.-] |
| 112 | K16348 | 4,644459529 | 1,458401431 | 0,432189648 | 3,374447857 | 0,00074 | 0,030277 | K16348 | ecnB | entericidin B |
| 10 | K00840 | 2,530889623 | 1,390033897 | 0,39263106 | 3,540305486 | 0,0004 | 0,020268 | K00840 | astC | succinylornithine aminotransferase [EC:2.6.1.81] |
| 132 | K19155 | 2,748952245 | 1,353785703 | 0,426057805 | 3,177469549 | 0,001486 | 0,049484 | K19155 | yhaV | toxin YhaV [EC:3.1.-.-] |
| 104 | K15836 | 2,553898745 | 1,343558248 | 0,375024368 | 3,582589187 | 0,00034 | 0,017897 | K15836 | fhlA | formate hydrogenlyase transcriptional activator |
| 11 | K00892 | 2,474300768 | 1,326314481 | 0,378997385 | 3,499534649 | 0,000466 | 0,02157 | K00892 | gsk | inosine kinase [EC:2.7.1.73] |
| 119 | K18146 | 2,287321885 | 1,302378152 | 0,407815416 | 3,193548116 | 0,001405 | 0,047655 | K18146 | adeB | multidrug efflux pump |
| 85 | K12288 | 2,359774868 | 1,275189677 | 0,383120144 | 3,328432858 | 0,000873 | 0,033318 | K12288 | hofM | pilus assembly protein HofM |
| 93 | K14054 | 2,972071054 | 1,238997862 | 0,386292293 | 3,20741026 | 0,001339 | 0,046253 | K14054 | mpaA | protein MpaA |
| 17 | K01643 | 1036,175927 | -1,103499948 | 0,254012005 | -4,344282656 | 1,40E-05 | 0,001633 | K01643 | citF | citrate lyase subunit alpha / citrate CoA-transferase [EC:2.8.3.10] |
| 18 | K01646 | 1026,291769 | -1,110431125 | 0,255815797 | -4,340744944 | 1,42E-05 | 0,001633 | K01646 | citD | citrate lyase subunit gamma (acyl carrier protein) |
| 22 | K02791 | 847,4185199 | -1,132356724 | 0,342211187 | -3,308941279 | 0,000936 | 0,034676 | K02791 | PTS-MalGlc-EIIC, malX | PTS system, maltose/glucose-specific IIC component |
| 19 | K02481 | 931,8789236 | -1,143326165 | 0,254878867 | -4,485762896 | 7,27E-06 | 0,001321 | K02481 | K02481 | two-component system, NtrC family, response regulator |
| 70 | K10714 | 2,775392778 | -1,230212037 | 0,386484419 | -3,18308314 | 0,001457 | 0,048823 | K10714 | mtdB | methylene-tetrahydromethanopterin dehydrogenase [EC:1.5.1.-] |
| 47 | K07288 | 171,8806856 | -1,244439061 | 0,352777016 | -3,527551409 | 0,000419 | 0,02053 | K07288 | tspA | uncharacterized membrane protein |
| 49 | K07312 | 3,272998552 | -1,244464335 | 0,373269796 | -3,333954015 | 0,000856 | 0,033011 | K07312 | ynfH | Tat-targeted selenate reductase subunit YnfH |
| 56 | K08298 | 3,891742077 | -1,280585851 | 0,399852297 | -3,202647227 | 0,001362 | 0,046552 | K08298 | caiB | L-carnitine CoA-transferase [EC:2.8.3.21] |
| 7 | K00696 | 3,426074434 | -1,363732785 | 0,411235307 | -3,31618604 | 0,000913 | 0,034245 | K00696 | E2.4.1.14 | sucrose-phosphate synthase [EC:2.4.1.14] |
| 50 | K07445 | 2,337733861 | -1,364206381 | 0,367183254 | -3,715328427 | 0,000203 | 0,013545 | K07445 | K07445 | putative DNA methylase |
| 98 | K15468 | 3,816459008 | -1,374483954 | 0,375771066 | -3,657769524 | 0,000254 | 0,015731 | K15468 | pksS | cytochrome P450 PksS |
| 116 | K17285 | 3,94568104 | -1,376836082 | 0,341693464 | -4,029448108 | 5,59E-05 | 0,004842 | K17285 | SELENBP1 | selenium-binding protein 1 |
| 75 | K11530 | 4,232867181 | -1,37859898 | 0,419356406 | -3,287416056 | 0,001011 | 0,036252 | K11530 | lsrG | (4S)-4-hydroxy-5-phosphonooxypentane-2,3-dione isomerase [EC:5.3.1.32] |
| 107 | K16163 | 5,256363637 | -1,391218168 | 0,43653474 | -3,186958655 | 0,001438 | 0,048462 | K16163 | K16163 | maleylpyruvate isomerase [EC:5.2.1.4] |
| 48 | K07310 | 3,132018869 | -1,400568083 | 0,372226844 | -3,762673504 | 0,000168 | 0,011828 | K07310 | ynfF | Tat-targeted selenate reductase subunit YnfF [EC:1.97.1.9] |
| 114 | K16873 | 5,225876479 | -1,417760554 | 0,395286238 | -3,586668133 | 0,000335 | 0,017786 | K16873 | hmfH | 5-(hydroxymethyl)furfural/furfural oxidase [EC:1.1.3.47 1.1.3.-] |
| 128 | K19064 | 3,361366808 | -1,424214443 | 0,407067776 | -3,498715758 | 0,000468 | 0,02157 | K19064 | lysDH | lysine 6-dehydrogenase [EC:1.4.1.18] |
| 8 | K00702 | 68,71695237 | -1,434559231 | 0,434378185 | -3,302558184 | 0,000958 | 0,034793 | K00702 | E2.4.1.20 | cellobiose phosphorylase [EC:2.4.1.20] |
| 15 | K01236 | 54,9275854 | -1,487489764 | 0,414240001 | -3,590888757 | 0,00033 | 0,017667 | K01236 | treZ, glgZ | maltooligosyltrehalose trehalohydrolase [EC:3.2.1.141] |
| 33 | K04105 | 5,547218787 | -1,487988553 | 0,414076551 | -3,593510789 | 0,000326 | 0,017658 | K04105 | hbaA | 4-hydroxybenzoate-CoA ligase [EC:6.2.1.27 6.2.1.25] |
| 51 | K07653 | 8,06450886 | -1,514948168 | 0,453908724 | -3,337561252 | 0,000845 | 0,033011 | K07653 | mprB | two-component system, OmpR family, sensor histidine kinase MprB [EC:2.7.13.3] |
| 52 | K07741 | 10,66761327 | -1,534264089 | 0,451868911 | -3,395374303 | 0,000685 | 0,028932 | K07741 | antB | anti-repressor protein |
| 110 | K16299 | 8,349403577 | -1,554762129 | 0,485553143 | -3,20204318 | 0,001365 | 0,046552 | K16299 | eexD | ATP-binding cassette, subfamily C, bacterial EexD |
| 67 | K10240 | 9,392437205 | -1,569994716 | 0,484001031 | -3,243783826 | 0,00118 | 0,041497 | K10240 | cebE | cellobiose transport system substrate-binding protein |
| 44 | K06376 | 7,497717591 | -1,582477817 | 0,487817286 | -3,243997009 | 0,001179 | 0,041497 | K06376 | spo0E | stage 0 sporulation regulatory protein |
| 86 | K12454 | 11,09724741 | -1,592230791 | 0,476737563 | -3,339847573 | 0,000838 | 0,033011 | K12454 | rfbE | CDP-paratose 2-epimerase [EC:5.1.3.10] |
| 26 | K03533 | 3,476860433 | -1,601637698 | 0,384848878 | -4,161731498 | 3,16E-05 | 0,003013 | K03533 | torD | TorA specific chaperone |
| 12 | K00960 | 7,45343245 | -1,604466257 | 0,482442493 | -3,325715046 | 0,000882 | 0,033318 | K00960 | E2.7.7.6 | DNA-directed RNA polymerase [EC:2.7.7.6] |
| 106 | K15980 | 3,309664278 | -1,61256908 | 0,405415113 | -3,977575152 | 6,96E-05 | 0,005763 | K15980 | ACAD9 | acyl-CoA dehydrogenase family member 9 [EC:1.3.99.-] |
| 95 | K14259 | 12,88526546 | -1,668350909 | 0,456531368 | -3,654405864 | 0,000258 | 0,015731 | K14259 | kdxD | 2-dehydro-3-deoxy-D-arabinonate dehydratase [EC:4.2.1.141] |
| 55 | K08253 | 4,060800359 | -1,66982225 | 0,377289267 | -4,425840851 | 9,61E-06 | 0,001321 | K08253 | E2.7.10.2 | non-specific protein-tyrosine kinase [EC:2.7.10.2] |
| 68 | K10242 | 10,04195353 | -1,676875781 | 0,474164175 | -3,536487719 | 0,000405 | 0,020379 | K10242 | cebG | cellobiose transport system permease protein |
| 25 | K03489 | 7,609476524 | -1,679957659 | 0,476226576 | -3,527643658 | 0,000419 | 0,02053 | K03489 | yydK | GntR family transcriptional regulator, transcriptional regulator of bglA |
| 103 | K15835 | 3,100581756 | -1,68159851 | 0,385704946 | -4,359805409 | 1,30E-05 | 0,001633 | K15835 | murR | RpiR family transcriptional regulator, murPQ operon repressor |
| 41 | K05599 | 2,445602454 | -1,696021683 | 0,37636986 | -4,506263289 | 6,60E-06 | 0,001321 | K05599 | antA | anthranilate 1,2-dioxygenase (deaminating, decarboxylating) large subunit [EC:1.14.12.1] |
| 74 | K10984 | 6,224857229 | -1,699476974 | 0,466805523 | -3,640653099 | 0,000272 | 0,016112 | K10984 | PTS-Gam-EIIB, agaB | PTS system, galactosamine-specific IIB component [EC:2.7.1.-] |
| 96 | K14338 | 5,198147751 | -1,725460177 | 0,402700828 | -4,284719717 | 1,83E-05 | 0,001972 | K14338 | cypD_E, CYP102A2_3 | cytochrome P450 / NADPH-cytochrome P450 reductase [EC:1.14.14.1 1.6.2.4] |
| 115 | K16929 | 10,19395983 | -1,7954872 | 0,518874954 | -3,460346633 | 0,000539 | 0,023724 | K16929 | K16929 | energy-coupling factor transport system substrate-specific component |
| 21 | K02615 | 4,202656845 | -1,817750172 | 0,417096679 | -4,358102721 | 1,31E-05 | 0,001633 | K02615 | paaJ | 3-oxo-5,6-didehydrosuberyl-CoA/3-oxoadipyl-CoA thiolase [EC:2.3.1.223 2.3.1.174] |
| 91 | K13810 | 11,16422922 | -1,828475776 | 0,435692544 | -4,196711192 | 2,71E-05 | 0,002722 | K13810 | tal-pgi | transaldolase / glucose-6-phosphate isomerase [EC:2.2.1.2 5.3.1.9] |
| 83 | K12081 | 9,690144441 | -1,834257791 | 0,523514586 | -3,503737702 | 0,000459 | 0,02152 | K12081 | ptlE | type IV secretion system protein PtlE |
| 5 | K00613 | 4,364008505 | -1,835548564 | 0,425768193 | -4,311145347 | 1,62E-05 | 0,001828 | K00613 | GATM | glycine amidinotransferase [EC:2.1.4.1] |
| 27 | K03658 | 15,13762989 | -1,836052044 | 0,489582879 | -3,750237445 | 0,000177 | 0,012277 | K03658 | helD | DNA helicase IV [EC:3.6.4.12] |
| 89 | K13735 | 9,695267833 | -1,849651301 | 0,499820488 | -3,700631215 | 0,000215 | 0,013915 | K13735 | yeeJ | adhesin/invasin |
| 118 | K18123 | 2,9179745 | -1,875937373 | 0,390322218 | -4,806125003 | 1,54E-06 | 0,00053 | K18123 | HOGA1 | 4-hydroxy-2-oxoglutarate aldolase [EC:4.1.3.16] |
| 29 | K03943 | 3,019052939 | -1,896518099 | 0,388799794 | -4,877878354 | 1,07E-06 | 0,000464 | K03943 | NDUFV2 | NADH dehydrogenase (ubiquinone) flavoprotein 2 [EC:1.6.5.3 1.6.99.3] |
| 4 | K00594 | 5,991872845 | -1,912435764 | 0,429775459 | -4,44984869 | 8,59E-06 | 0,001321 | K00594 | xyoA, aldO | alditol oxidase [EC:1.1.3.41] |
| 105 | K15924 | 16,14599364 | -1,9375504 | 0,53451533 | -3,624873394 | 0,000289 | 0,016112 | K15924 | xynC | glucuronoarabinoxylan endo-1,4-beta-xylanase [EC:3.2.1.136] |
| 109 | K16216 | 12,02631374 | -1,984633342 | 0,509641847 | -3,894172652 | 9,85E-05 | 0,007367 | K16216 | yueD | benzil reductase ((S)-benzoin forming) [EC:1.1.1.320] |
| 88 | K13409 | 56,31465767 | -1,992645224 | 0,524283248 | -3,80070359 | 0,000144 | 0,010548 | K13409 | raxB, cvaB | ATP-binding cassette, subfamily B, bacterial RaxB |
| 38 | K05519 | 6,707848753 | -2,005292975 | 0,47398795 | -4,230683449 | 2,33E-05 | 0,002406 | K05519 | med | transcriptional activator of comK gene |
| 123 | K18481 | 6,736163504 | -2,068080993 | 0,489058844 | -4,228695624 | 2,35E-05 | 0,002406 | K18481 | mas | Mce-associated membrane protein |
| 97 | K14647 | 9,831839803 | -2,118423233 | 0,507281083 | -4,176034359 | 2,97E-05 | 0,002879 | K14647 | vpr | minor extracellular serine protease Vpr [EC:3.4.21.-] |
| 140 | K19789 | 14,4513677 | -2,194856154 | 0,445140175 | -4,930707854 | 8,19E-07 | 0,000384 | K19789 | radD | DNA repair protein RadD |
| 108 | K16190 | 4,042296514 | -2,253782305 | 0,408053703 | -5,523249235 | 3,33E-08 | 2,34E-05 | K16190 | GLCAK | glucuronokinase [EC:2.7.1.43] |
| 13 | K00972 | 22,98913991 | -2,288660909 | 0,501932531 | -4,559698307 | 5,12E-06 | 0,001109 | K00972 | UAP1 | UDP-N-acetylglucosamine/UDP-N-acetylgalactosamine diphosphorylase [EC:2.7.7.23 2.7.7.83] |
| 60 | K09000 | 49,86079588 | -2,30572926 | 0,58488465 | -3,942194859 | 8,07E-05 | 0,006312 | K09000 | cmr4 | CRISPR-associated protein Cmr4 |
| 62 | K09127 | 49,86079588 | -2,30572926 | 0,58488465 | -3,942194859 | 8,07E-05 | 0,006312 | K09127 | cmr3 | CRISPR-associated protein Cmr3 |
| 130 | K19141 | 49,86079588 | -2,30572926 | 0,58488465 | -3,942194859 | 8,07E-05 | 0,006312 | K19141 | cmr5 | CRISPR-associated protein Cmr5 |
| 16 | K01342 | 7,992468895 | -2,325392059 | 0,483308696 | -4,81140124 | 1,50E-06 | 0,00053 | K01342 | aprE | subtilisin [EC:3.4.21.62] |
| 72 | K10954 | 199,2588092 | -2,366297905 | 0,589005533 | -4,01744597 | 5,88E-05 | 0,004943 | K10954 | zot | zona occludens toxin |
| 6 | K00693 | 5,021788361 | -2,378730084 | 0,40555632 | -5,865350794 | 4,48E-09 | 6,31E-06 | K00693 | GYS | glycogen synthase [EC:2.4.1.11] |
| 131 | K19145 | 50,46585821 | -2,505889144 | 0,547411842 | -4,577703574 | 4,70E-06 | 0,001058 | K19145 | csx16 | CRISPR-associated protein Csx16 |
| 134 | K19272 | 48,03465068 | -2,522550645 | 0,578073468 | -4,363719811 | 1,28E-05 | 0,001633 | K19272 | aph3-I | aminoglycoside 3'-phosphotransferase I [EC:2.7.1.95] |
| 14 | K01169 | 48,04702731 | -2,527023698 | 0,564829966 | -4,473954727 | 7,68E-06 | 0,001321 | K01169 | rna | ribonuclease I (enterobacter ribonuclease) [EC:3.1.27.6] |
| 124 | K18909 | 4,722480058 | -2,541752195 | 0,412832785 | -6,156856453 | 7,42E-10 | 1,96E-06 | K18909 | mepR | MarR family transcriptional regulator, repressor for mepA |
| 45 | K07061 | 47,72041654 | -2,562844935 | 0,575800338 | -4,450926416 | 8,55E-06 | 0,001321 | K07061 | cmr1 | CRISPR-associated protein Cmr1 |
| 129 | K19076 | 47,72041654 | -2,562844935 | 0,575800338 | -4,450926416 | 8,55E-06 | 0,001321 | K19076 | cmr2, cas10 | CRISPR-associated protein Cmr2 |
| 63 | K09228 | 47,65979553 | -2,570836318 | 0,575758696 | -4,465128073 | 8,00E-06 | 0,001321 | K09228 | KRAB | KRAB domain-containing zinc finger protein |
| 101 | K15640 | 7,515176238 | -2,620780506 | 0,472235272 | -5,549734763 | 2,86E-08 | 2,33E-05 | K15640 | K15640, phoE | uncharacterized phosphatase |
| 92 | K13829 | 43,95219693 | -2,645828767 | 0,459115405 | -5,762883877 | 8,27E-09 | 9,31E-06 | K13829 | aroKB | shikimate kinase / 3-dehydroquinate synthase [EC:2.7.1.71 4.2.3.4] |
| 135 | K19427 | 11,71589886 | -3,344727349 | 0,475235184 | -7,038046548 | 1,95E-12 | 1,10E-08 | K19427 | epsJ | glycosyltransferase EpsJ [EC:2.4.-.-] |

Table 3 suppl. DESeq2-identified differentially abundant PICRUSt2-predicted KO functions encoded by

genomes of bacteria thriving in saliva samples.
