# Supplementary material for: The oral cavity and intestinal microbiome in children with functional constipation
Source: Sci Rep. 2024 Apr 9;14:8283. doi: 10.1038/s41598-024-58642-2 (PMC11004141; doi:10.1038/s41598-024-58642-2)
Supplement: Supplementary file 4 — Supplementary Table 4. [file 41598_2024_58642_MOESM4_ESM.docx]

|  | Row.names | baseMean | log2FoldChange | lfcSE | stat | pvalue | padj | fun | desc |
| --- | --- | --- | --- | --- | --- | --- | --- | --- | --- |
| 9 | PWY-5531 | 18,85163941 | 3,579225982 | 0,589235265 | 6,074358061 | 1,24E-09 | 1,49E-07 | PWY-5531 | chlorophyllide a biosynthesis II (anaerobic) |
| 11 | PWY-7159 | 18,85163941 | 3,579225982 | 0,589235265 | 6,074358061 | 1,24E-09 | 1,49E-07 | PWY-7159 | chlorophyllide a biosynthesis III (aerobic, light independent) |
| 4 | METHGLYUT-PWY | 14,16704828 | 3,571647875 | 0,568283098 | 6,284979943 | 3,28E-10 | 1,18E-07 | METHGLYUT-PWY | superpathway of methylglyoxal degradation |
| 2 | ARGDEG-PWY | 5,014218925 | 2,750516342 | 0,48624137 | 5,656689272 | 1,54E-08 | 1,11E-06 | ARGDEG-PWY | superpathway of L-arginine, putrescine, and 4-aminobutanoate degradation |
| 5 | ORNARGDEG-PWY | 5,014218925 | 2,750516342 | 0,48624137 | 5,656689272 | 1,54E-08 | 1,11E-06 | ORNARGDEG-PWY | superpathway of L-arginine and L-ornithine degradation |
| 8 | PWY-5265 | 4,304621845 | 2,570632255 | 0,516992903 | 4,972277647 | 6,62E-07 | 3,97E-05 | PWY-5265 | peptidoglycan biosynthesis II (staphylococci) |
| 7 | PWY-3661 | 21,84735434 | 2,391041198 | 0,585717631 | 4,082242146 | 4,46E-05 | 0,001784 | PWY-3661 | glycine betaine degradation I |
| 10 | PWY-6629 | 3,221933734 | 2,051599305 | 0,478737911 | 4,285433128 | 1,82E-05 | 0,000821 | PWY-6629 | superpathway of L-tryptophan biosynthesis |
| 6 | ORNDEG-PWY | 3,169175307 | 2,021175981 | 0,453820488 | 4,453690472 | 8,44E-06 | 0,000434 | ORNDEG-PWY | superpathway of ornithine degradation |
| 1 | 3-HYDROXYPHENYLACETATE-DEGRADATION-PWY | 11,45921344 | 1,883347695 | 0,507635623 | 3,710038477 | 0,000207 | 0,00746 | 3-HYDROXYPHENYLACETATE-DEGRADATION-PWY | 4-hydroxyphenylacetate degradation |
| 3 | ECASYN-PWY | 3,668072759 | 1,434367899 | 0,427911759 | 3,352017957 | 0,000802 | 0,022216 | ECASYN-PWY | enterobacterial common antigen biosynthesis |
| 12 | PWY-7347 | 4,754175764 | -1,677362892 | 0,456442842 | -3,674858576 | 0,000238 | 0,007788 | PWY-7347 | sucrose biosynthesis III |
| 13 | SUCSYN-PWY | 12,91170395 | -2,037570082 | 0,568058208 | -3,586903687 | 0,000335 | 0,010039 | SUCSYN-PWY | sucrose biosynthesis I (from photosynthesis) |

Table 4 suppl. DESeq2-identified differentially abundant PICRUSt2-predicted pathways encoded by

genomes of bacteria thriving in saliva samples.
