# Supplementary material for: The oral cavity and intestinal microbiome in children with functional constipation
Source: Sci Rep. 2024 Apr 9;14:8283. doi: 10.1038/s41598-024-58642-2 (PMC11004141; doi:10.1038/s41598-024-58642-2)
Supplement: Supplementary file 5 — Supplementary Table 5. [file 41598_2024_58642_MOESM5_ESM.docx]

| Parameter | Children  with FC  n= 46 (100%) | Children without FC  n= 25 (100%) | P |
| --- | --- | --- | --- |
| Fiber intake, n (%)   - below the recommended value - within the norm - above the recommended value | 24 (52.2)  3 (6.5)  19 (41.3) | 8 (32)  6 (24)  11 (44) | >0.05 |
| Water consumption, n (%)   - values below normal - values within the norm - values above normal | 9 (19.6)  7 (15.2)  30 (65.2) | 6 (25.0)  8 (33.3)  10 (41.7) | >0.05 |
| Fat consumption, n (%)   - values below normal - values within the norm - values above normal | 25 (54.3)  19 (41.3)  2 (4.3) | 14 (56)  11 (44)  0 (0.0) | >0.05 |
| Protein consumption, n (%)   - values within the norm - values above normal | 35 (76.1)  11 (23.9) | 18 (72)  7 (28) | >0.05 |
| Carbohydrate consumption, n (%)   - values below normal - values within the norm - values above normal | 2 (4.3)  42 (91.3)  2 (4.3) | 0 (0.0)  25 (100)  0 (0.0)) | >0.05 |
| Kilocalories intake, n (%)   - values below normal - values within the norm - values above normal | 27 (58.7)  12 (26.1)  7 (15.2) | 15 (60)  6 (24)  4 (16) | >0.05 |
| Magnesium intake- RDA*, n (%)   - below the recommended value - within the norm - above the recommended value | 11 (23.9)  6 (13.0)  29 (63) | 6 (24)  4 (16)  15 (60) | >0.05 |
| Calcium intake- RDA, n (%)   - below the recommended value - within the norm - above the recommended value | 36 (80)  5 (11.1)  4 (8.9) | 25 (100)  0 (0.0)  0 (0.0) | >0.05 |

Table 1 Suppl. Quantitative and qualitative diet composition in children with FC vs children without FC.

FC- functional constipation; RDA- recommended daily allowance
