# Supplementary material for: The oral cavity and intestinal microbiome in children with functional constipation
Source: Sci Rep. 2024 Apr 9;14:8283. doi: 10.1038/s41598-024-58642-2 (PMC11004141; doi:10.1038/s41598-024-58642-2)
Supplement: Supplementary file 6 — Supplementary Table 6. [file 41598_2024_58642_MOESM6_ESM.docx]

| Parameter | Children with FC  n= 57 (100%) | Children without FC  n= 34 (100%) | P |
| --- | --- | --- | --- |
| How many times per week does your child engage in physical activity, n (%)   - does not exercise - one time per week - two- three times per week - more than three times per week | 5 (8.8)  3 (5.3)  23 (40.4)  26 (45.6) | 1 (2.9)  8 (23.5)  13 (38.2)  12 (35.3) | >0.05 |
| Does the child participate in additional sports activities, n (%)   - yes - no | 18 (31.6)  39 (68.4) | 8 (23.5)  26 (76.5) | >0.05 |
| Does the child willingly engage in physical activity, n (%)   - willingly - is persuaded by parents | 43 (75.4)  14 (24.6) | 31 (91.2)  3 (8.8) | >0.05 |
| Time spent by the child in front of the computer, n (%)   - less than 1 hour a day - 1-3 hours a day - more than 1 hour a day | 26 (45.6)  27 (47.4)  4 (7) | 18 (52.9)  14 (41.2)  2 (5.9) | >0.05 |
| Time spent by the child in front of the TV,  n (%)   - less than 1 hour a day - 1-3 hours a day - more than 1 hour a day | 36 (63.2)  20 (35.1)  1 (1.8) | 21 (61.8)  13 (38.2)  0 (0.0) | >0.05 |

Table 2 Suppl. Physical activity in children with FC vs children without FC.
